# TSLRF: Two-Stage Algorithm Based on Least Angle Regression and Random Forest in genome-wide association studies

**DOI:** 10.1038/s41598-019-54519-x

**Published:** 2019-12-02

**Authors:** Jiali Sun, Qingtai Wu, Dafeng Shen, Yangjun Wen, Fengrong Liu, Yu Gao, Jie Ding, Jin Zhang

**Affiliations:** College of Science, Nanjing Agricultural University, Nanjing, 210095 China

**Keywords:** Genome-wide association studies, Agricultural genetics

## Abstract

One of the most important tasks in genome-wide association analysis (GWAS) is the detection of single-nucleotide polymorphisms (SNPs) which are related to target traits. With the development of sequencing technology, traditional statistical methods are difficult to analyze the corresponding high-dimensional massive data or SNPs. Recently, machine learning methods have become more popular in high-dimensional genetic data analysis for their fast computation speed. However, most of machine learning methods have several drawbacks, such as poor generalization ability, over-fitting, unsatisfactory classification and low detection accuracy. This study proposed a two-stage algorithm based on least angle regression and random forest (TSLRF), which firstly considered the control of population structure and polygenic effects, then selected the SNPs that were potentially related to target traits by using least angle regression (LARS), furtherly analyzed this variable subset using random forest (RF) to detect quantitative trait nucleotides (QTNs) associated with target traits. The new method has more powerful detection in simulation experiments and real data analyses. The results of simulation experiments showed that, compared with the existing approaches, the new method effectively improved the detection ability of QTNs and model fitting degree, and required less calculation time. In addition, the new method significantly distinguished QTNs and other SNPs. Subsequently, the new method was applied to analyze five flowering-related traits in *Arabidopsis*. The results showed that, the distinction between QTNs and unrelated SNPs was more significant than the other methods. The new method detected 60 genes confirmed to be related to the target trait, which was significantly higher than the other methods, and simultaneously detected multiple gene clusters associated with the target trait.

## Introduction

With the rapid development of biotechnology and sequencing technology, a large number of high-dimensional genetic data have been generated. How to analysis these kind datasets is a hot topic. The pervasive feature of genetic data is hundreds of thousands single-nucleotide polymorphisms (SNPs) along with a few hundred or thousand samples, that is, the “big P, small N” problem, which brings challenges to data mining and statistical analysis^1–3^.

However, a number of studies illustrate that most quantitative traits are controlled by a small portion of genetic markers among all SNPs^4–6^. Variable selection is the process of detecting the subset of potential variables associating with the phenotype, thus avoiding intractable problems of high-dimensional datasets analysis. There are many variable selection methods proposed for “big P, small N” datasets. The least absolute shrinkage and selection operator^7^ (LASSO) method minimizes the residual sum of squares, subject to the sum of the absolute value of the coefficients being less than a constant, which has the advantages of subset selection and ridge regression. Since LASSO is hard to implement and often has a high false positive rate in the detection^8^, many extension methods were continuously proposed under the framework of variable selection, such as, least angle regression^9^ (LARS), the smoothly clipped absolute deviation (SCAD) penalized^10^ method, the elastic net^11^ method and the adaptive LASSO^12^ method. Although most of the mythologies are successfully applied to genome-wide association analysis (GWAS) analysis^13–16^, they still suffer from the high false positive rate in detection, which hard to provide theoretical basis for the following work, say, fine mapping and gene cloning.

Machine learning methods are alternative to classical statistical approaches of mining “big P, small N” genetic datasets by optimizing the classification. Support vector machine^17^ (SVM) is applied to reprioritize GWAS results^5^; artificial neural network^18^ (ANN) is utilized to classify and select quantitative trait nucleotides (QTNs) associating with complex traits in genome-wide association data^4^.

Random forest^19^ (RF) has become a popular machine learning method in recent years. In RF, multiple decision trees are constructed by autonomous sampling (Bagging) and node random splitting technique, the final classification or regression results are obtained by voting or averaging. RF is easy to implement and has relatively fast learning speed. Recently, the improved RF methods have been proposed and applied to GWAS, Botta *et al*.^20^ and Nguyen *et al*.^21^ respectively proposed T-Trees method and ts-RF method, both of them optimize the split rules of RF decision tree nodes; Szymczak *et al*.^22^ redefined the calculation method of importance scores; Elyan & Gaber^23^ optimized the setting of *ntree* and *mtry*; Stephan *et al*.^24^ proposed a hybrid RF algorithm. However, most of the existing methods do not consider the population structure and polygenetic effect background, and the importance scores of all variables are maintained at the same level, so it is hard to distinguish whether the variable is related to the target trait or not.

In real data analysis at present, the methodologies for genome-wide single-marker scan under polygenic background and population structure controls are widely used to conduct GWAS, such as efficient mixed model association (EMMA)^25^ and its improved method EMMA eXpedited (EMMAX)^26^, which reduces the computational time for analyzing large GWAS datasets from years to hours. On the other hand, multi-locus GWAS analysis methods have also been proposed, such as fast multi-locus random-SNP-effect EMMA (FASTmrEMMA)^16^, which is more powerful in QTN detection and model fit.

In this study, we proposed a two-stage association analysis method by combining variable selection with machine learning, two-stage algorithm based on least angle regression and random forest (TSLRF). TSLRF firstly adopted the model transformation of FASTmrEMMA^16^ method to control the population structure and correct the polygenic background, secondly applied LARS to select the subset of variables, which were potentially related to the target traits, finally utilized RF to calculate the importance scores and rankings of selected SNPs from LARS. The new method could flexibly scan each SNPs from the high dimensional datasets by constructing a fast and new matrix transformation. Furtherly, TSLRF improved the importance scores of QTNs, which were associated with target traits, thus boosted the signals of related SNPs.

We conducted the simulation experiments (199 individuals, 10,000 SNPs) and real *Arabidopsis thaliana* data analysis (derived from 199 individuals, 216,130 SNPs, which are over 1,000 times larger than sample size) to validate the reliability of the new method. All the results showed that, TSLRF is relatively superior to the other five methods including classical RF in model fitting degree, prediction accuracy and computing time, it can effectively increase the discrimination between QTNs and SNPs. In addition, for real *Arabidopsis* data, the number of confirmed genes which are detected by TSLRF is significantly more than the other methods.

## Materials and Methods

### Genetic model

Let *y*_*i*_(*i* = 1, 2, …, *n*) be the phenotypic observation value of the *i*th individual in a natural population with *n* samples. The genetic model can be described as:

1 $${\boldsymbol{y}}=W{\boldsymbol{\alpha }}+Z{\boldsymbol{\gamma }}+{\bf{u}}+{\boldsymbol{\varepsilon }}$$

where ${\boldsymbol{y}}={({y}_{1},\mathrm{...},{y}_{n})}^{T}$; ***α*** is a c × 1 vectorthe intercept, population structure effect and so on; ${\boldsymbol{\gamma }} \sim MV{N}_{p}({\bf{0}},\,{{\boldsymbol{\Sigma }}}_{{\boldsymbol{\gamma }}})$ is random effect of putative QTN; ${{\boldsymbol{\Sigma }}}_{{\boldsymbol{\gamma }}}=diag\{{{\sigma }_{1}}^{2},\mathrm{...},{{\sigma }_{p}}^{2}\}$; *p* is the number of putative QTNs; **W** and **Z** are the corresponding designed matrices for **α** and **γ**; ${\boldsymbol{u}} \sim MV{N}_{n}(0,\,{{\sigma }_{g}}^{2}{\boldsymbol{K}})$ is a random $n\times 1$ vector of polygenic effects; **K** is a known $n\times n$ relatedness matrix; ${\boldsymbol{\varepsilon }}$ is residual error with an assumed $MV{N}_{n}(0,{\sigma }^{2}{{\boldsymbol{I}}}_{n})$ distribution; ${\sigma }^{2}$ is residual error variance; ***I***_*n*_ is an $n\times n$ identity matrix; $MV{N}_{n}({\boldsymbol{\mu }},{\boldsymbol{\Sigma }})$ denotes multivariate normal distribution with *n*-dimensional mean vector ***u*** and $n\times n$ covariance matrix ${\boldsymbol{\Sigma }}$. To control polygenic background, The model transformation^16^ is adapted to whiten the covariance matrix of the polygenic matrix ***K*** and residual noise. By decomposing the variance of ***y*** in the model (1) and standardizing designed matrices for fixed and random effects (Supplementary methods), model (1) can be rewritten as:

2 $${\boldsymbol{Y}}=X{\boldsymbol{\beta }}+{\boldsymbol{\varepsilon }}$$

where ***Y*** is phenotype after corrected; **X** is marker genotype after standardized, where $\mathop{\sum }\limits_{i=1}^{n}{x}_{ij}=0$, $\mathop{\sum }\limits_{i=1}^{n}{{x}_{ij}}^{2}=1(j=1,\,2,\,\mathrm{...},\,p)$; $${\boldsymbol{\beta }}=(\begin{array}{c}{\boldsymbol{\alpha }}\\ {\boldsymbol{\gamma }}\end{array})$$ is a vector of fixed and random effects; ${\boldsymbol{\varepsilon }}$ is residual error with an assumed $MV{N}_{n}(0,{\sigma }^{2}{{\boldsymbol{I}}}_{n})$ distribution.Here, we integrated LARS and RF to implement GWAS in simulation study and real data analysis. LARS algorithm overcomes the deficiency in calculation of LASSO, and it is an effective approach for computing and analyzing high-dimensional data. Here, LARS selected the variables having the largest absolute correlation with the response ***y***, say *x*_*j1*_, and performed simple linear regression between them to calculate residual vector which was orthogonal to ${x}_{{j}_{1}}\,\,$and now considered to be the response, then projected the other variables orthogonal to ${x}_{{j}_{l}}$ and repeated the selection process. The details of LARS algorithm are shown in Supplementary methods.

As LARS terminates at the saturated least squares fit after *n-*1 steps, where *n−*1 variables have entered the active set, a LARS fit always has no more than *n−*1 variables with nonzero coefficients^9^. Efron *et al*.^9^ provided a *Cp*-type risk estimation formula of a *s*-step LARS estimator ${\hat{{\boldsymbol{\mu }}}}_{s}$ as:

3 $${{\boldsymbol{C}}}_{{\boldsymbol{p}}}({\hat{{\boldsymbol{\mu }}}}_{s})=\frac{{\Vert {\boldsymbol{Y}}-{\hat{{\boldsymbol{\mu }}}}_{s}\Vert }^{2}}{{\bar{\sigma }}^{2}}-n+2s$$

where, ${\bar{\sigma }}^{2}$ is the usual ordinary least squares (OLS) estimates of ${\sigma }^{2}$ from the full OLS model.

The smaller *Cp* indicates the better LARS estimation. We applied LARS to analyzing one simulated dataset which were randomly selected from 1,000 replication simulated datasets, and obtained the *Cp* values and residual sum of squares (RSS) values of ***Y*** and ${\hat{{\boldsymbol{\mu }}}}_{s}$ at each step (Supplementary Fig. S1). It clearly shows that, as the iteration steps increases, the value of *Cp* and *RSS* decrease. At the maximum iteration step, $s=n-1$ step, the model performs best with the smallest *Cp*, 196, and the smallest *RSS*, 0.0137. For the other replications, the results have the same trend. Therefore, we set the maximum iteration step *s* as *n-*1 in simulation experiments and real *Arabidopsis* data analyses, then at most *n-*1 variables which were most likely to be associated with the target traits could be selected to construct a RF model. In the following section, we recorded the number of variables selected by LARS as *k*.

In order to select potentially significant variable set, the selected potential variable set $\{{x}_{{j}_{1}},{x}_{{j}_{2}},\ldots ,{x}_{{j}_{k}}\}$ by LARS was constructed to the RF model. RF first used Bagging algorithm to generate *ntree* (default is 500 in RF) new self-sampling sample sets and *ntree* out of bag (OOB) datasets from the original training dataset. Then, a RF model was constructed by *ntree* CART trees generated by node random splitting technique, which randomly extracted *mtry* variables from the *k* variables at each node of each tree. Here, we set *mtry* be the value that could make the RF model get the best model fitting degree. RF took the average output of all CART trees as the prediction results in regression. The details of conducting RF are shown in Supplementary methods. Finally, we calculated the importance scores of the *k* selected variables by the variable importance measure (VIM) method in RF. VIM calculated the importance scores by OOB error rate: *%IncMSE*, which was obtained by randomly replacing the value of the calculated variable to form new OOB test data and calculating its prediction error. The larger *%IncMSE* indicates the variable more important. The details of calculating variable importance scores are shown in Supplementary methods. According to the importance scores, we could rank *k* variables and select the set of potentially significant variables furtherly.

TSLRF is an R-based implementation, where achieved LARS and RF using the package *lars* and *randomForest*, respectively, in R program.

### Evaluation indicators

In this study, mean absolute error (*MAE*), mean absolute percentage error (*MAPE*) and Pearson correlation coefficient *r* were selected to evaluate the performance of the new method.

***MAE*** is defined as:

4 $$MAE=\frac{1}{n}\mathop{\sum }\limits_{i=1}^{n}|{y}_{i}-{\hat{y}}_{i}|$$

*MAE* is often used to assess the divergence between predicted and true value. The larger *MAE* indicates the lower accuracy of the results, on the contrary, the smaller the better. Assuming that ${y}_{i}(i=1,\,2,\,\ldots ,\,n)$ is the phenotypic observations of quantitative traits, ${\hat{y}}_{i}(i=1,\,2,\,\ldots ,\,n)$ is the predicted value from ten-fold cross validation, the following symbols have the same meaning.

***MAPE*** is defined as:

5 $$MAPE=\frac{1}{n}\mathop{\sum }\limits_{i=1}^{n}|\frac{{y}_{i}-\widehat{{y}_{i}}}{{y}_{i}}|$$

different from *MAE*, *MAPE* considers the deviation between predicted and true values, moreover, it takes the proportion between deviation and true values into account. In this way, the error will be placed under the unified scale, and the predictions will be more accurate. *MAPE* is also one of the commonly used objective functions in some competitions. Like *MAE*, the smaller *MAPE* the better.

Pearson correlation coefficient ***r*** is defined as:

6 $$r=\mathop{\sum }\limits_{i=1}^{n}\,(\frac{{y}_{i}-\bar{y}}{{s}_{y}})\,(\frac{{\hat{y}}_{i}-\bar{\hat{y}}}{{s}_{\hat{y}}})$$

where, $\bar{y}=\frac{1}{n}\,\mathop{\sum }\limits_{i=1}^{n}{y}_{i}$, $\bar{\hat{y}}=\frac{1}{n}\,\mathop{\sum }\limits_{i=1}^{n}{\hat{y}}_{i}$, ${s}_{y}=\sqrt{\frac{{\sum }_{i=1}^{n}{({y}_{i}-\bar{y})}^{2}}{n-1}}$, ${s}_{\hat{y}}=\sqrt{\frac{{\sum }_{i=1}^{n}{({\hat{y}}_{i}-\bar{\hat{y}})}^{2}}{n-1}}$; Pearson correlation coefficient |r| ≤ 1, indicating the degree of proximity between the predicted value and the true value. The larger absolute value of r indicates the predicted value closer to the true value.

### Experimental materials

In this study, the performance of the TSLRF method was verified by using the simulation datasets and *Arabidopsis* real datasets. Each dataset contained phenotypic observations of quantitative traits and SNP markers, and the number of SNP variables was hundreds or thousands of times larger than the sample size.

We simulated 1,000 datasets (replications) with 199 individuals and 10,000 SNPs derived from *Arabidopsis* natural population^27^, all of the SNPs were spaced on five chromosome segments, the positions of these SNPs in the genome were between 11,226,256 and 12,038,776 bp on Chr. 1, between 5,045,828 and 6,412,875 bp on Chr. 2, between 1,916,588 and 3,196,442 bp on Chr. 3, between 2,232,796 and 3,143,893 bp on Chr. 4 and between 19,999,868 and 21,039,406 bp on Chr. 5^28^. The simulation datasets have been analyzed by several studies^6,16,28^ previously. Six QTNs were simulated and placed on the SNPs with allelic frequencies of 0.30. Their heritabilities were set as 0.10, 0.05, 0.05, 0.15, 0.05 and 0.05, respectively. The heritability here is the narrow sense heritability^29^, whose value is equal to the proportion of breeding value variance to phenotypic variance, which also indicates the coefficient of determination *r*^2^ for breeding value and phenotypic value. The heritability and position of each QTN are listed on Table 1. The total average and residual variance were both set at 10.0. Here, 1,000 simulation datasets (replications) were analyzed by several established methods and the new method TSLRF.

**Table 1:** The comparison of TSLRF, TSRF and RF in the simulation experiment.

QTN	Chr.	Position	r^2^(%)^#^	Importance score		
				TSLRF	TSRF	RF
1	1	11298364	10	8.240 (3.5068)*	3.849 (2.1362)	0.974 (0.9176)
2	1	11655607	5	4.998 (2.6696)	2.084 (2.1669)	0.662 (0.9459)
3	2	5066968	5	5.349 (2.7481)	2.196 (2.1439)	0.667 (0.9695)
4	2	5134228	15	9.851 (3.0699)	4.594 (1.6362)	1.222 (0.7815)
5	2	5464675	5	5.157 (2.3644)	1.905 (2.0249)	0.688 (0.9324)
6	2	6137189	5	4.266 (2.4887)	1.515 (2.1581)	0.586 (0.9323)

^*^The values in parentheses were the standard deviation of the importance scores obtained in 1,000 replication simulation analyses for each QTN.

^#^The heritability of simulated QTNs.

The real dataset included 199 *Arabidopsis* lines (http://www.arabidopsis.usc.edu/) with 216,130 SNPs and 107 traits^27^. Among these traits, we analyzed five traits related to flowering time, including LD: days to flowering under long days; LDV: days to flowering under long days with vernalization; SD: days to flowering under short days; FT16: days to flowering at 16C; FT22: days to flowering at 22C. These data were downloaded from the following website: http://www.arabidopsis.usc.edu/.

## Results

### Analysis of the simulated datasets

To validate the performance of TSLRF, the simulation experiment was implemented, firstly. We generated 1,000 datasets (replications) from *Arabidopsis* inbreed population, and simulated six QTNs, all of which were placed at SNP positions. All the simulation information is listed on Table 1 as described above. Each of the 1,000 replications was analyzed by TSLRF, two-stage stepwise variable selection based on random forest^30^(TSRF), RF, support vector regression (SVR), ANN and EMMAX, respectively. Subsequently, we used three statistical indicators mentioned above: *MAE*, *MAPE* and *r* to evaluate the prediction accuracy and model fitting of the above six approaches. Meanwhile, we also compared the importance scores of three RF methods (TSLRF, TSRF and RF) and the computing time of six methods. All the six methods are R-based implementation, where three RF framework methods are implemented using *randomForest* package; SVR and ANN are implemented using *e1071* and *nnet* packages, respectively; EMMAX is implemented using Genomic Association and Prediction Integrated Tool (GAPIT) software.

### Model accuracy

**Figure 1:**
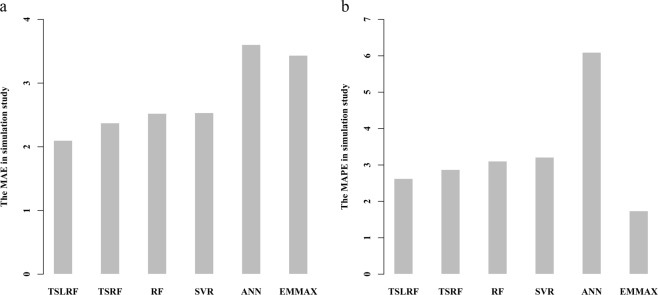
The mean absolute error (*MAE*, panel a) and the mean absolute percentage error (*MAPE*, panel b) of ten-fold cross-validation in 1,000 repeated simulated analyses by using the two-stage algorithm based on least angle regression and random forest (TSLRF), two-stage stepwise variable selection based on random forests (TSRF), random forest (RF), support vector regression (SVR), artificial neural network (ANN) and EMMA eXpedited (EMMAX).

The model accuracy of the six methods were evaluated by the ten-fold cross validation. The *MAE* and *MAPE* of 1,000 repeated simulated data analyses by six methods are shown on Supplementary Table S1. The Fig. 1a illustrates the *MAE* of the TSLRF, TSRF, RF, SVR, ANN and EMMAX, as it shows, TSLRF has the smallest *MAE*, 2.0997, which indicates that TSLRF is more accurate among them; the *MAE* of TSRF, RF and SVR are similar to each other, they are 2.3745, 2.5236 and 2.5345, respectively; the *MAE* of EMMAX and ANN are 3.4358 and 3.6041, larger than the other four methods. Obviously, ANN has the lowest model accuracy among these five methods.

The *MAPE* of TSLRF, TSRF, RF, SVR and ANN (Fig. 1b) has the same trend as *MAE*. Differently, the *MAPE* of EMMAX is 1.7393, which is smaller than TSLRF, 2.6270, the tendency is contrast to *MAE*.

These two measurements show that, among the six approaches, TSLRF performs relatively better model accuracy than the other established methods, the performance of ANN is not satisfied.

### Importance scores

**Figure 2:**
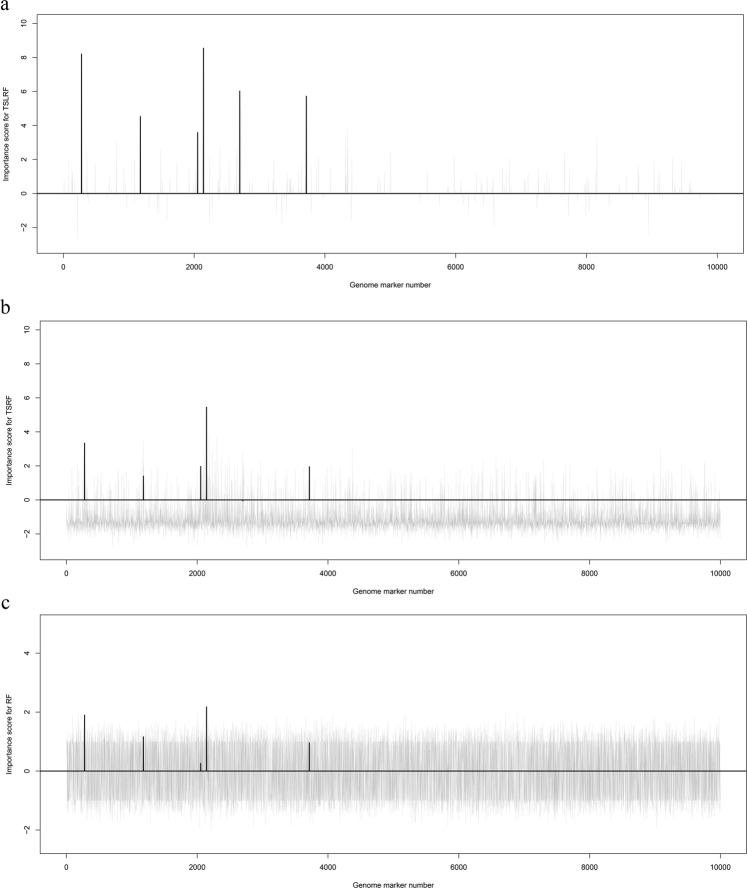
Importance scores of 10,000 SNPs of one replication dataset (from 1,000 replication simulated datasets) analysis by using TSLRF (panel a), TSRF (panel b) and RF (panel c).

For the three methods (TSLRF, TSRF and RF) under the RF framework, we detected whether SNPs were related to the target traits through ranking their importance scores. We randomly selected the result of one simulation dataset (from 1,000 replications) for analysis, the importance scores obtained from the above three methods are shown in Fig. 2. The gray needles indicate the estimated importance scores of the corresponding SNPs, and the black thick needles represent the estimated importance scores of the simulated QTNs related to the target trait. The results of the TSLRF (Fig. 2a) show that the six simulated QTNs located on chromosome 1 and chromosome 2 have higher importance scores and rankings, making it easy to distinguish QTNs and other SNPs. However, the importance scores and rankings of six simulated QTNs computed by TSRF (Fig. 2b) and RF (Fig. 2c) do not perform well.

Meanwhile, we calculated the average importance scores of 1,000 repeated simulated analyses for three RF methods. As shown on Table 2, for TSLRF, the importance score of top-ranked QTN was 9.851, which was more than two times as much as TSRF (4.594) and nine times much than RF (1.222). The average importance score of the six simulated QTNs also has the same trend, 6.310 in TSLRF, 2.690 in TSRF and 0.800 in RF. However, in three RF methods, the average scores of other SNPs unrelated to target trait were all less than 1. Obviously, compared to TSRF and RF, TSLRF significantly improves the ability to distinguish QTNs and SNPs.

**Table 2:** The average important scores of top-ranked QTN, six simulated QTNs and unrelated SNPs respectively, calculated by TSLRF, TSRF and RF methods in 1,000 replicated simulated analyses.

Importance score	TSLRF	TSRF	RF
top-ranked QTN	9.851	4.594	1.222
Average of six simulated QTNs	6.310	2.690	0.800
Average of unrelated SNPs	0.852	−1.166	0.006

In addition, Table 1 and Supplementary Fig. S2 show the average importance scores in 1,000 replicated simulated data analyses of each QTN. It can be clearly seen that the QTN with a larger heritability tends to have a more significant importance score. As shown on Table 1, QTN4 located in chromosome 2 with 15% hereditability had the highest importance score (9.851) among all of the QTNs, importance score of QTN1 with 10% hereditability followed QTN4 (8.240), the rest QTNs with the same hereditability had similar scores, about 5.

We also analyzed the simulation datasets using SVR, ANN and EMMAX. For SVR and ANN, we obtained the weight of each SNP; for EMMAX, the effect of each SNP. Based on weights or effects, the importance rankings of all SNPs can be easily obtained. However, the weights, effects and the importance scores are not under the unified scale, so we did not list the weights or effects obtained by SVR, ANN and EMMAX here.

### Higher degree of model fitting

We measured the model fitting degree by calculating Pearson correlation coefficient *r* between the true phenotypic value and the predicted value, which was calculated by the *predict* function in R program. The average *r* for 1,000 replications calculated by TSLRF, TSRF, RF, SVR, ANN and EMMAX are shown on Supplementary Table S1. Clearly, the model fitting degree in TSLRF, TSRF, RF, SVR and EMMAX are at the same level, larger than 0.90, which means that all of the five methods have dramatic higher model fitting degree than ANN. SVR has the highest *r*, TSLRF follows it, which has the higher *r* among the three RF methods.

### Fast computing time

**Figure 3:**
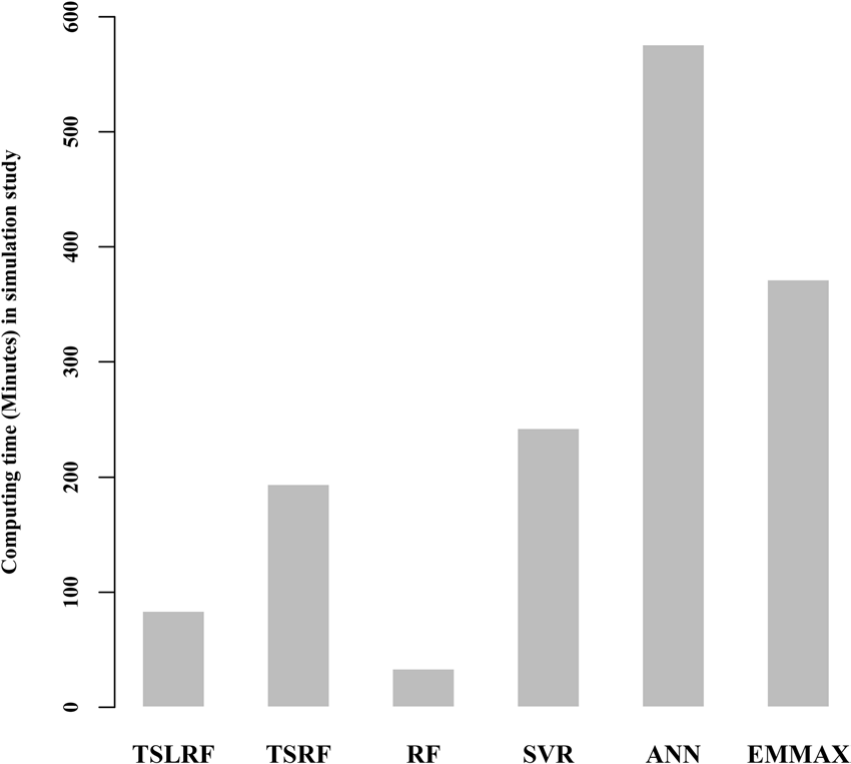
Computing time (minutes) of 1,000 repeated simulated analyses using TSLRF, TSRF, RF, SVR, ANN and EMMAX.

We compared the computing time of 1,000 repeated simulated analyses using six approaches. Figure 3 shows that, the three approaches under RF framework have fast computing speed than the other three methods. For 1,000 replications, the computing time of TSLRF and RF were on the same order of magnitude, only cost less 100 minutes, say, less 6 seconds for one replication averagely, followed by TSRF, SVR and EMMAX. ANN took the most expensive computing time about 600 minutes, which was nearly 7 times more than TSLRF.

For the model accuracy and the computing time, the performance of ANN was unsatisfied, thus, the ANN method was not used for analyzing the *Arabidopsis* datasets in the next section.

### Analysis of the *arabidopsis* datasets

The well-known *Arabidopsis* data^27^ includes 199 diverse inbred lines, each of which has 216,130 SNPs and 107 traits. To verify the performance of TSLRF, we re-analyzed five traits related to flowering time, including LD, LDV, SD, FT16 and FT22, and compared the result of TSLRF with that of TSRF, RF, SVR and EMMAX. The SNPs with minor allele frequency (MAF) less than 10% were deleted, the population structure and polygenic background were controlled by the model transformation of FASTmrEMMA^6,16^ (in the package *mrMLM* in R program). All the SNPs within 20 KB of each putative QTN were used to mine the candidate genes by TAIR (https://www.arabidopsis.org/) for these five traits. The detected putative QTNs associated with interested traits were used to fit the regression for each trait and model fitness was assessed by the indicators as above.

Table 3 showed the quantity of detected genes (confirmed by TAIR) using five approaches. For TSLRF, the numbers of detected genes which were significantly related (top 20) to the five traits (LD, LDV, SD, FT16 and FT22) were 10, 11, 16, 13 and 10 (Table 3), respectively, and the total quantity of confirmed genes was 60. The corresponding numbers of the associated genes was 52 for TSRF, 37 for RF, 40 for SVR and 39 for EMMAX. Apparently, for TSLRF, the quantity of significant genes (confirmed by TAIR) related to the interested flowering traits, is much larger than the other methods.

**Table 3:** The number of confirmed genes (top 20) detected by four methods (TSLRF, TSRF, RF, SVR and EMMAX) under five traits (LD, LDV, SD, FT16 and FT22) in analysis of *Arabidopsis* natural population.

Methods	LD	LDV	SD	FT16	FT22	Total
TSLRF	10	11	16	13	10	60
TSRF	5	10	12	8	17	52
RF	8	8	4	10	7	37
SVR	7	6	16	4	7	40
EMMAX	6	10	12	7	4	39

LD: days to flowering under long days; LDV: days to flowering under long days with vernalization; SD: days to flowering under short days; FT16: days to flowering at 16C; FT22: days to flowering at 22C.

Interestingly, the new method dissected several clusters of genes, which were associated with the same trait (Supplementary Table S2), such as, gene AT2G06990, AT2G07020, AT2G07040 and AT2G07050, adjacent to the SNP located at 2,916,675 bp on chromosome 2, were found relating to short days (SD); gene AT2G07020, AT2G07040 and AT2G07050, adjacent to the SNP located at 2,924,501 bp on chromosome 2, were confirmed as well as associating with SD. More importantly, several genes could be simultaneously detected by multiple traits, say gene AT3G50870 could be simultaneously detected under LD, SD and FT22. Moreover, Supplementary Table S3 shows that, for TSLRF, many genes detected associating with target traits could be simultaneously detected by other methods, for example, gene AT2G06990, AT2G07020, AT2G07040 and AT2G07050, adjacent to the SNP located at 2,916,675 bp and 2,910,430 bp on chromosome 2, which were confirmed associating with SD, could be simultaneously detected by TSLRF, TSRF and SVR. It demonstrates that TSLRF is more efficient and accurate than the other four methods in terms of detection capabilities.

In addition, the genes (confirmed by TAIR) detected by TSLRF obviously owned higher rankings compared with the other four methods (Supplementary Table S3). Take LD as an example, for TSLRF, the confirmed gene with the highest ranking was gene AT2G22540 (adjacent to the SNP located at 9,588,685 bp on chromosome 2), which ranked 1st; however, in TSRF, RF, SVR and EMMAX, the genes with the highest ranking were gene AT3G13530, AT1G03457, AT5G06500 and AT5G01180, ranked 4th, 3rd, 3rd and 5th, respectively. It means that top 2, 3 or 4 SNPs detected by these methods seem to be less important from the perspective of confirmed gene by TAIR. For the other traits (LDV, SD, FT16 and FT22), the rankings also have the same tendency. As clearly shown on Table 3, for the proposed method TSLRF, the total quantity of confirmed genes (ranked top 20) was much more than the other four methods. All of the above evidences show that, TSLRF can efficiently distinguish QTNs and SNPs, and has accurate detection capability.

**Figure 4:**
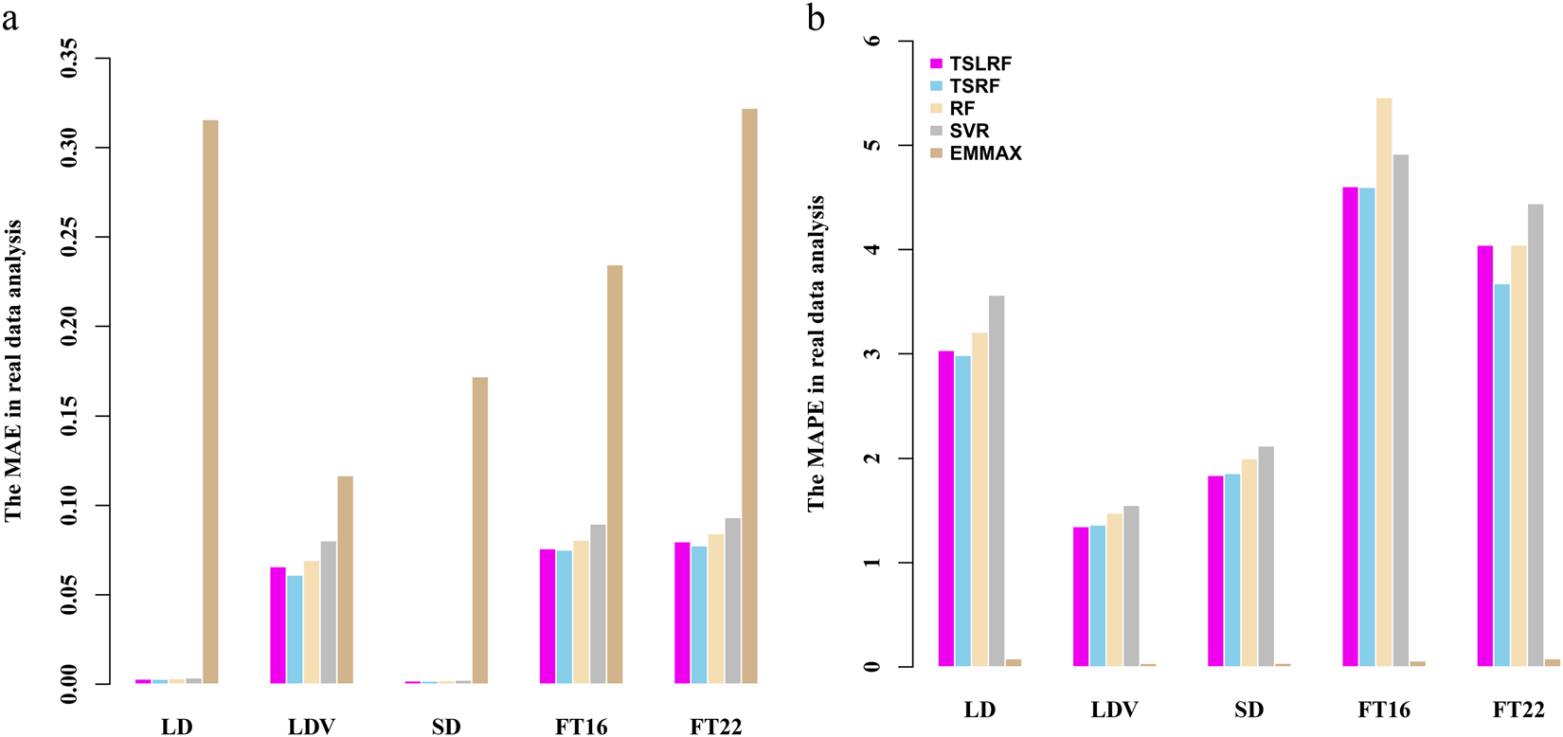
The *MAE* (panel a) and *MAPE* (panel b) of ten-fold cross validation results of TSLRF, TSRF, RF, SVR and EMMAX in analysis of *Arabidopsis* natural population.

We implemented ten-fold cross validation to evaluate the model accuracy. Figure 4 illustrates that, for TSLRF, the level of model accuracy (*MAE* and *MAPE*) is similar to that of TSRF, which is more accurate than RF and SVR, EMMAX performs well than TSLRF in terms of *MAPE*. Moreover, Supplementary Table S4 shows that, TSLRF, TSRF, RF, SVR and EMMAX also have high model fitting degrees which are at the same level, where, SVR and EMMAX are slightly better, TSLRF following them, has better model fitting degree among the three RF framework methods.

The computing time (Table 4) for high-dimensional *Arabidopsis* genetic datasets (216,130 SNPs, 199 individuals) using five methods indicates that, TSLRF, RF and SVR have similar computing time, and the data analysis time of each trait is about 2 minutes. EMMAX takes about 15 minutes to analyze a trait. Unlike simulation experiment, TSRF spend more than 60 times longer than TSLRF.

**Table 4:** Computing time (minutes) of analysis for *Arabidopsis* natural population using TSLRF, TSRF, RF, SVR and EMMAX.

Methods	TSLRF	TSRF	RF	SVR	EMMAX
LD	1.350	63.872	0.859	1.675	15.026
LDV	1.352	74.366	0.864	1.596	14.910
SD	1.273	70.552	0.815	1.531	14.287
FT16	1.688	85.678	1.014	2.029	14.613
FT22	1.672	76.027	1.010	2.071	14.459

## Discussion

In recent years, GWAS has become an important method to dissect complexity traits. A number of studies demonstrate that controlling population structure and kinship indeed improves the power of detection and decreases the false discover^25,28,31,32^. In this paper, we proposed a new method called two-stage algorithm based on least angle regression and random forest (TSLRF), which adopted the model transformation of FASTmrEMMA^16^, this background control algorithm whitens the covariance matrix of the polygenic matrix **K** and environmental noise, and specifies the number of nonzero eigenvalues as one. All the results in this paper show that, population structure and polygenic background control are crucial for TSLRF in GWAS, particularly for the importance rankings and scores of SNPs.

Variable selection plays a critical role in TSLRF, here LARS was applied in TSLRF to select the markers most likely associated with the target trait. To obtain accurate results, we using a simulated dataset which were randomly selected from 1,000 replication simulated datasets to compare different variable selection cases, setting the maximum iteration step of LARS from 1 to $n-1$, and found that, when the maximum iteration step was set as $n-1$, the model performs best with the smallest *Cp* and *RSS*. Then, we used at most $n-1$ selected variables to build subsequent RF model. Finally, the results of the selected top 20 potential related SNPs turned out the stability of TSLRF.

For the computing time, the new method TSLRF and classical RF both perform better and have faster speed than the other four methods (TSRF, SVR, ANN and EMMAX), that is the reason of RF so popular in machine learning. TSRF, as a method under the RF framework, takes quite expensive computing time (more than one hour) in real data analysis (Table 4), which is due to the fact that, it first grouping the original data and uses RF to calculate the importance scores, removes the last variable set with the lowest importance scores; secondly, calculates the importance scores of the remaining variables, and deletes the last variable set with the lowest importance scores; finally, repeats the above process until the last group completes their calculation. This kind of repeated sampling greatly increases the computing time. In the simulation datasets, the number of SNPs was just 10,000, which was divided into only 10 groups with 1,000 SNPs a group, the repeated sampling here did not obviously increase the calculation time, so it still performed a relatively fast computing speed. However, for the *Arabidopsis* real datasets, large-scale datasets resulted in computing time increasing exponentially. Therefore, although TSRF increases the distinction of QTNs and SNPs, the proposed new method, TSLRF, is recommended from the point of computing time.

## Supplementary information

Supplementary Information

## Data Availability

All data generated or analyzed during this study are included in this published article.
