# Supplementary material for: TSLRF: Two-Stage Algorithm Based on Least Angle Regression and Random Forest in genome-wide association studies
Source: Sci Rep. 2019 Dec 2;9:18034. doi: 10.1038/s41598-019-54519-x (PMC6889171; doi:10.1038/s41598-019-54519-x)
Supplement: Supplementary file 1 — Supplementary Information [file 41598_2019_54519_MOESM1_ESM.pdf]

TSLRF: Two-stage genome-wide association analysis method based on massive gene markers

Jiali Sun[1], Qingtai Wu[1], Dafeng Shen[1], Yangjun Wen[1], Fengrong Liu[1], Yu Gao[1], Jie Ding[1] and Jin Zhang[1,*]

[1] College of Science, Nanjing Agricultural University, Nanjing 210095, China.

*zhangjin@njau.edu.cn

List of included materials:

Supplementary Figure S1, S2

Supplementary Tables S1, S2, S3, S4

Supplementary Methods

Supplementary References

Supplementary Software S1

Supplementary Figures

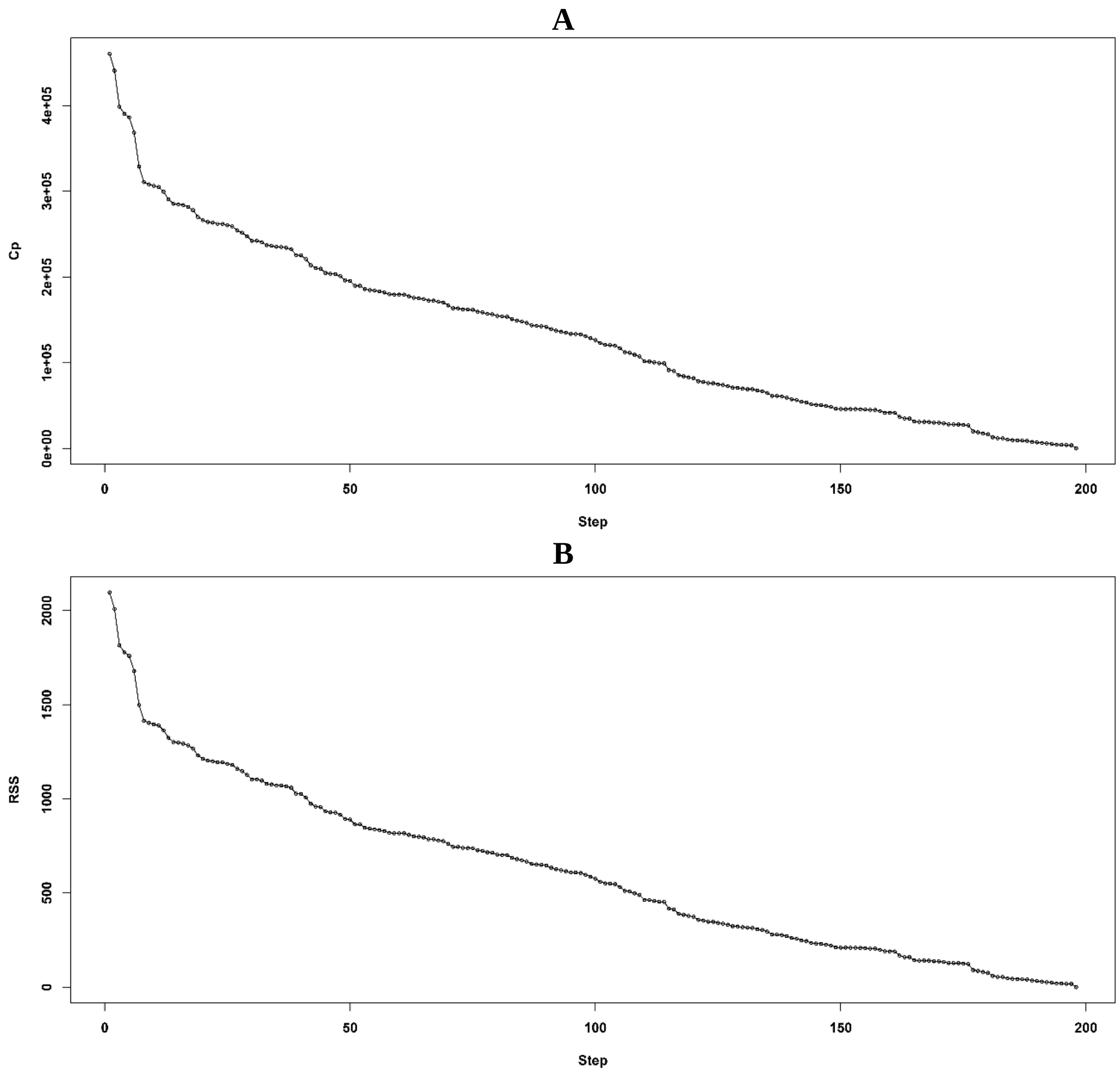

Figure S1: The risk estimation statistic *Cp* (A) and residual sum of squares (RSS, B) at each least angle regression (LARS) iteration step.

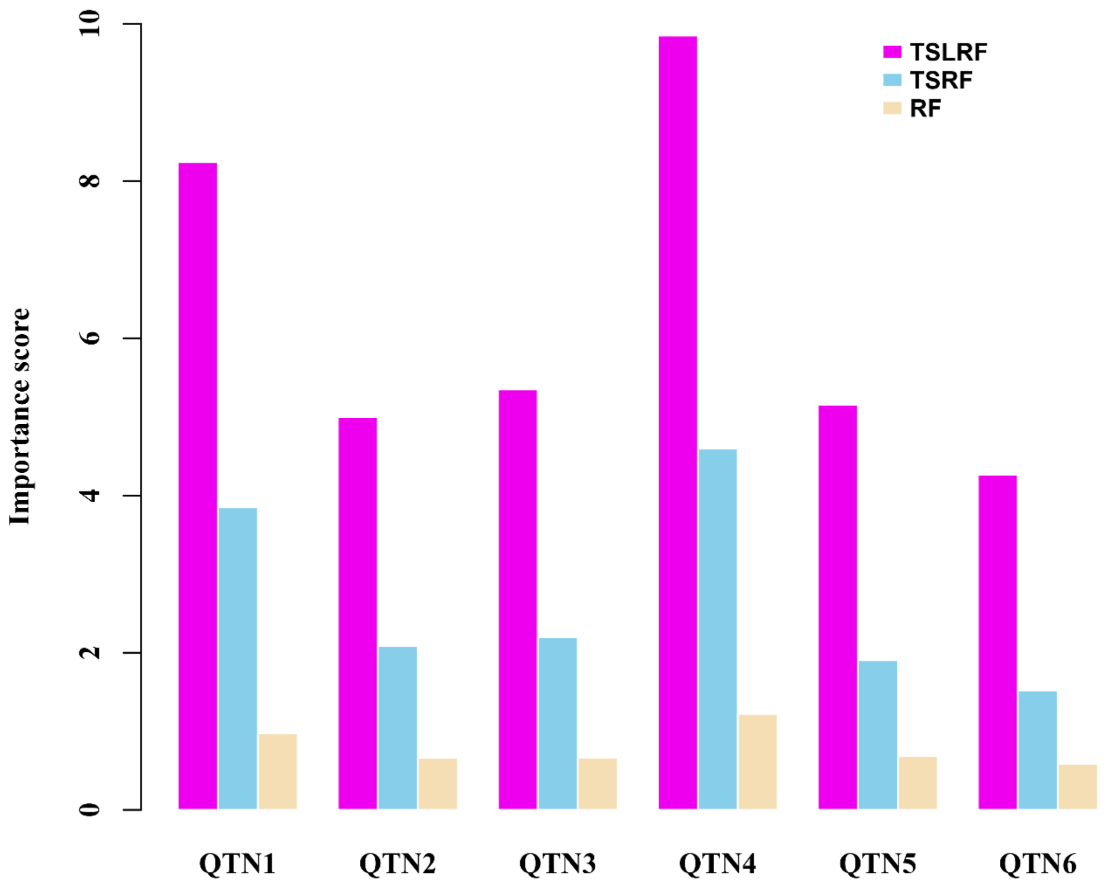

Figure S2: The average importance scores of 1,000 replicated simulated analyses of each QTN, using two-stage algorithm based on least angle regression and random forest (TSLRF), two-stage stepwise variable selection based on random forests (TSRF) and random forest (RF).

Supplementary tables

Table S1: The mean absolute error (*MAE*), mean absolute percentage error (*MAPE*) and Pearson coefficient *r* of ten-fold cross-validation in 1,000 repeated simulated analyses using TSLRF, TSRF, RF, support vector regression (SVR), artificial neural network (ANN) and EMMA eXpedited (EMMAX).

Method	MAE	MAPE	*r*
TSLRF	2.0997	2.6270	0.9936
TSRF	2.3745	2.8733	0.9923
RF	2.5236	3.1052	0.9919
SVR	2.5345	3.2127	0.9982
ANN	3.6041	6.0949	0.7930
EMMAX	3.4358	1.7393	0.9229

Table S2: The confirmed genes (top 20) using TSLRF under five traits (LD, LDV, SD, FT16 and FT22) in *Arabidopsis* natural population.

Trait	Chr.	Position	Gene	Importance score	Rank	Chr.	Position	Gene	Importance score	Rank
LD	2	9588685	AT2G22540	7.461	1	5	1993499	AT5G06500	4.208	13
	3	10855475	AT3G28860	5.807	2	1	1661549	AT1G05580	4.184	14
	5	3188328	AT5G10140	4.913	6	3	18923922	AT3G50870	4.151	15
	1	8051936	AT1G22770	4.902	7	5	2305726	AT5G07280	4.016	16
	4	429928	AT4G00990	4.291	12	5	25407568	AT5G63470	3.98	18
LDV	1	10700018	AT1G30330	7.515	1	5	8248050	AT5G24240	3.542	13
	4	518797	AT4G01220	5.064	3	2	8516520	AT2G19690	3.428	16
	2	9606045	AT2G22630	4.56	6			AT2G19760	3.428	16
	5	18590678	AT5G45840	4.415	8	3	18055603	AT3G48750	3.38	17
	3	18068801	AT3G48750	4.235	9	4	17025163	AT4G35900	3.331	19
	4	14002571	AT4G28190	3.631	12					
SD	3	18923922	AT3G50870	5.665	2	1	3744308	AT1G11130	3.404	15
	3	10855475	AT3G28860	5.385	4			AT1G11190	3.404	15
	5	1993499	AT5G06500	4.933	5	2	2924501	AT2G07020	3.398	16
	2	2916675	AT2G06990	3.84	10			AT2G07040	3.398	16
			AT2G07020	3.84	10			AT2G07050	3.398	16
			AT2G07040	3.84	10	5	18627646	AT5G45890	3.268	18
			AT2G07050	3.84	10	3	15189648	AT3G43210	3.129	20
	4	153402	AT4G00315	3.439	13					
			AT4G00330	3.439	13					
FT16	3	18959000	AT3G51060	5.31	1	5	8307127	AT5G24330	4.529	9
	3	23090917	AT3G62440	4.828	2	5	3188328	AT5G10140	4.424	10
	3	18953585	AT3G51060	4.721	4	4	529188	AT4G01220	4.008	14
	3	18960953	AT3G51060	4.706	5	4	11166402	AT4G20910	3.907	16
	3	18964911	AT3G51060	4.554	6	1	10700018	AT1G30330	3.893	17
	2	9236317	AT2G21540	4.548	7	4	519513	AT4G01220	3.651	19
	3	18068801	AT3G48750	4.537	8					
FT22	3	23090917	AT3G62440	6.841	1	4	409692	AT4G00990	3.801	12
	3	18960737	AT3G51060	5.789	2	5	14772437	AT5G37260	3.725	13
	3	18923922	AT3G50870	5.539	4	4	625569	AT4G01500	3.706	14
	5	2305726	AT5G07280	5.351	5	1	9488653	AT1G27320	3.469	19
	1	9491094	AT1G27320	4.093	9					
			AT1G27380	4.093	9					

LD: days to flowering under long days; LDV: days to flowering under long days with vernalization; SD: days to flowering under short days; FT16: days to flowering at 16C; FT22: days to flowering at 22C.

Table S3: The confirmed genes (top 20) detected by TSLRF, TSRF, RF, SVR and EMMAX under five flowering time traits (LD, LDV, SD, FT16, FT22) in *Arabidopsis* natural population.

Trait	Gene	Chr.	Position	Rank	Method	Gene	Chr.	Position	Rank	Method
**LD**	AT1G01030	1	6096	13	EMMAX	AT3G13530	3	4412299	4	TSRF
	AT1G01040	1	40689	11	EMMAX	AT3G28860	3	10855475	2	TSLRF
	AT1G01040	1	6096	13	EMMAX	AT3G44880	3	16381547	16	SVR
	AT1G01060	1	40689	11	EMMAX	AT3G50870	3	18923922	15	TSLRF
	AT1G03457	1	882633	3	RF	**AT4G00990**	4	429928	12	TSLRF
	**AT1G05580**	1	1661549	14	TSLRF	**AT4G00990**	4	419382	12	RF
				7	SVR	AT4G35900	4	16991753	15	RF
	AT1G11410	1	3841346	7	TSRF	AT5G01180	5	43661	5	EMMAX
	AT1G22770	1	8051936	7	TSLRF	**AT5G06500**	5	1993499	13	TSLRF
	AT1G30330	1	10700018	17	SVR				3	SVR
	AT1G45050	1	17029170	20	RF	AT5G07280	5	2305726	16	TSLRF
	AT1G68540	1	25721229	4	RF	AT5G07530	5	2402568	9	SVR
	AT2G01820	2	340279	14	RF	AT5G07530	5	2403991	12	SVR
	AT2G22540	2	9588685	1	TSLRF	AT5G10140	5	3188328	6	TSLRF
	AT2G27300	2	11675923	18	TSRF	AT5G12330	5	3999439	17	RF
	AT2G31910	2	13589731	13	TSRF	AT5G12370	5	3999439	17	RF
	AT3G01040	3	25341	8	EMMAX	AT5G24860	5	8540425	11	SVR
	AT3G12810	3	4088981	19	TSRF	AT5G63470	5	25407568	18	TSLRF
**LDV**	AT1G05580	1	1661549	3	SVR	AT3G62980	3	23294580	18	SVR
	AT1G06170	1	1888680	16	TSRF	AT3G63010	3	23290445	4	SVR
	AT1G06180	1	1888680	16	TSRF	AT3G63010	3	23294580	18	SVR
	AT1G06220	1	1888680	16	TSRF	AT3G63480	3	23431943	16	RF
	AT1G07340	1	2260699	9	TSRF	AT4G01220	4	518797	3	TSLRF
	**AT1G13400**	1	4597161	20	TSRF	AT4G28190	4	14002571	12	TSLRF
	**AT1G13400**	1	4606174	10	RF	AT4G35900	4	17025163	19	TSLRF
	AT1G28270	1	9892280	14	TSRF	AT5G01180	5	41108	3	EMMAX
	**AT1G30330**	1	10700018	1	TSLRF	AT5G01180	5	43959	5	EMMAX
				1	SVR	AT5G01180	5	43467	6	EMMAX
	AT1G47260	1	17327833	18	RF	AT5G01180	5	41024	8	EMMAX
	AT1G47270	1	17327833	18	RF	AT5G01180	5	43402	10	EMMAX
	AT1G59640	1	21900098	12	TSRF	AT5G01180	5	41962	11	EMMAX
	AT1G73177	1	27513366	17	TSRF	AT5G01180	5	41112	13	EMMAX
	AT2G19690	2	8516520	16	TSLRF	AT5G01180	5	41047	15	EMMAX
	AT2G19760	2	8516520	16	TSLRF	AT5G01180	5	43129	16	EMMAX
	**AT2G22630**	2	9606045	6	TSLRF	AT5G01180	5	43826	20	EMMAX
	**AT2G22630**	2	9637537	17	RF	AT5G12210	5	3960111	2	RF
	AT3G01780	3	302012	11	TSRF	AT5G24240	5	8248050	13	TSLRF
	AT3G01890	3	302012	11	TSRF	AT5G45840	5	18590678	8	TSLRF
	AT3G48750	3	18068801	9	TSLRF	AT5G55300	5	22442708	9	RF
	AT3G48750	3	18055603	17	TSLRF	AT5G55390	5	22442708	9	RF
	AT3G62980	3	23290445	4	SVR					

Trait	Gene	Chr.	Position	Rank	Method	Gene	Chr.	Position	Rank	Method
**SD**	AT1G01030	1	5620	6	EMMAX	**AT2G07020**	2	2917760	6	SVR
	AT1G01030	1	5042	9	EMMAX	**AT2G07020**	2	2896216	9	SVR
	AT1G01030	1	5047	10	EMMAX	**AT2G07020**	2	2910430	20	TSRF
	AT1G01030	1	3822	15	EMMAX	**AT2G07040**	2	2916675	10	TSLRF
	AT1G01030	1	5037	17	EMMAX				7	SVR
	AT1G01030	1	4731	20	EMMAX	**AT2G07040**	2	2924501	16	TSLRF
	AT1G01040	1	40294	5	EMMAX	**AT2G07040**	2	2917760	6	SVR
	AT1G01040	1	46736	7	EMMAX	**AT2G07040**	2	2910430	20	TSRF
	AT1G01060	1	40294	5	EMMAX	**AT2G07050**	2	2916675	10	TSLRF
	AT1G01060	1	46736	7	EMMAX				7	SVR
	AT1G06040	1	1823369	6	RF	**AT2G07050**	2	2924501	16	TSLRF
	AT1G08320	1	2637910	14	TSRF	**AT2G07050**	2	2917760	6	SVR
	AT1G08390	1	2637910	14	TSRF	**AT2G07050**	2	2910430	20	TSRF
	AT1G09730	1	3152601	15	TSRF	AT2G22540	2	9578960	17	SVR
	AT1G09780	1	3152601	15	TSRF	AT2G47040	2	19335027	3	RF
	AT1G11130	1	3744308	15	TSLRF	AT3G01040	3	25125	4	EMMAX
	AT1G11190	1	3744308	15	TSLRF	AT3G01890	3	305078	9	TSRF
	AT1G11410	1	3844044	8	TSRF	AT3G28860	3	10855475	4	TSLRF
	AT1G11410	1	3832974	11	TSRF	AT3G43210	3	15189648	20	TSLRF
	AT1G22770	1	8083571	15	SVR				20	SVR
	AT1G28520	1	10043763	18	TSRF	AT3G50870	3	18923922	2	TSLRF
	AT1G53160	1	19820034	10	SVR	AT3G51060	3	18950155	3	SVR
	**AT2G06990**	2	2916675	10	TSLRF	AT4G00315	4	153402	13	TSLRF
				7	SVR	AT4G00330	4	153402	13	TSLRF
	**AT2G06990**	2	2917760	6	SVR	AT5G01180	5	75401	12	EMMAX
	**AT2G06990**	2	2896216	9	SVR	**AT5G06500**	5	1993499	5	TSLRF
	**AT2G06990**	2	2910430	20	TSRF				1	SVR
	**AT2G07020**	2	2916675	10	TSLRF	AT5G24080	5	8126252	19	RF
				7	SVR	AT5G45890	5	18627646	18	TSLRF
	**AT2G07020**	2	2924501	16	TSLRF	AT5G59030	5	23851738	8	RF
**FT16**	AT1G01030	1	5960	12	EMMAX	AT3G51060	3	18959000	1	TSLRF
	AT1G01040	1	5960	12	EMMAX	AT3G51060	3	18953585	4	TSLRF
	AT1G01060	1	52864	1	EMMAX	AT3G51060	3	18960953	5	TSLRF
	AT1G01060	1	52911	2	EMMAX	AT3G51060	3	18964911	6	TSLRF
	AT1G01060	1	52969	3	EMMAX	AT3G60460	3	22347505	19	RF
	AT1G01060	1	52914	11	EMMAX	AT3G62440	3	23090917	2	TSLRF
	AT1G09730	1	3135281	17	TSRF	AT3G62980	3	23294580	6	SVR
	AT1G18450	1	6369609	7	TSRF	AT3G63010	3	23294580	6	SVR
	**AT1G30330**	1	10700018	17	TSLRF	AT4G01220	4	529188	14	TSLRF
				3	SVR	AT4G01220	4	519513	19	TSLRF
	AT1G49490	1	18308112	15	TSRF	AT4G20910	4	11166402	16	TSLRF
	AT1G52740	1	19629918	19	TSRF	AT4G22140	4	11734214	9	RF
	**AT2G21540**	2	9236317	7	TSLRF	AT5G05850	5	1750051	14	RF
				13	SVR	AT5G06070	5	1837286	17	RF
	AT2G44010	2	18226445	2	RF	AT5G06100	5	1837286	17	RF
	AT3G01040	3	25063	7	EMMAX	AT5G06110	5	1837286	17	RF
	AT3G01780	3	284291	4	TSRF	AT5G10140	5	3188328	10	TSLRF
	AT3G11440	3	3601213	6	TSRF	AT5G24330	5	8307127	9	TSLRF
	AT3G11450	3	3601213	6	TSRF	AT5G46795	5	18998754	4	RF
	AT3G48750	3	18068801	8	TSLRF	AT5G53390	5	21672548	3	RF
	AT3G50870	3	18929030	3	TSRF	AT5G53400	5	21672548	3	RF

Trait	Gene	Chr.	Position	Rank	Method	Gene	Chr.	Position	Rank	Method
**FT22**	AT1G01040	1	46155	19	EMMAX	AT3G05000	3	1367585	15	TSRF
	AT1G01060	1	46155	19	EMMAX	AT3G11440	3	3599162	8	TSRF
	AT1G05580	1	1661549	18	SVR	AT3G11450	3	3599162	8	TSRF
	AT1G06040	1	1824924	12	TSRF	AT3G12680	3	4043914	10	TSRF
	AT1G27320	1	9491094	9	TSLRF	AT3G12690	3	4043914	10	TSRF
	AT1G27320	1	9488653	19	TSLRF	AT3G15170	3	5102027	4	SVR
	AT1G27380	1	9491094	9	TSLRF	AT3G50870	3	18923922	4	TSLRF
	AT1G27600	1	9614653	20	TSRF	**AT3G51060**	3	18960737	2	TSLRF
	AT1G27650	1	9614653	20	TSRF	**AT3G51060**	3	18959000	1	TSRF
	AT1G30330	1	10700018	6	SVR	**AT3G62440**	3	23090917	1	TSLRF
	AT1G63820	1	23671053	11	RF	**AT3G62440**	3	23079300	12	SVR
	AT1G75520	1	28371242	2	RF	AT3G62980	3	23294580	10	SVR
	AT1G80680	1	30329214	9	TSRF	AT3G63010	3	23294580	10	SVR
	AT2G06990	2	2914102	11	TSRF	AT4G00650	4	267418	3	RF
	AT2G07020	2	2914102	11	TSRF	AT4G00990	4	409692	12	TSLRF
	AT2G07040	2	2914102	11	TSRF	AT4G01500	4	625569	14	TSLRF
	AT2G07050	2	2914102	11	TSRF	AT4G36920	4	17413519	8	RF
	AT2G21540	2	9236317	14	SVR	AT4G36930	4	17413519	8	RF
	AT2G21870	2	9309635	16	RF	AT5G01180	5	41808	17	EMMAX
	AT2G22370	2	9497667	16	TSRF	AT5G07280	5	2305726	5	TSLRF
	AT2G37040	2	15579004	7	TSRF	AT5G37020	5	14616833	19	RF
	AT2G46340	2	19021144	13	TSRF	AT5G37260	5	14772437	13	TSLRF
	AT3G01040	3	25054	1	EMMAX					

The gene labeled as blue bold are the genes that can be detected by more than one method at the same time.

Table S4: The *MAE*, *MAPE* and Pearson coefficient *r* of ten-fold cross-validation using TSLRF, TSRF, RF, SVR and EMMAX in *Arabidopsis* natural population.

Trait		TSLRF	TSRF	RF	SVR	EMMAX
LD	MAE	2.95E-03	2.84E-03	3.14E-03	3.60E-03	3.16E-01
	MAPE	3.0333	2.9850	3.2076	3.5639	0.0792
	r	0.9917	0.9890	0.9884	0.9987	0.9999
LDV	MAE	6.58E-02	6.11E-02	6.92E-02	8.03E-02	1.17E-01
	MAPE	1.3472	1.3629	1.4763	1.5514	0.0348
	r	0.9882	0.9863	0.9863	0.9978	0.9896
SD	MAE	1.89E-03	1.77E-03	2.00E-03	2.31E-03	1.72E-01
	MAPE	1.8386	1.8565	1.9983	2.1207	0.0365
	r	0.9912	0.9894	0.9890	0.9984	0.9999
FT16	MAE	7.59E-02	7.51E-02	8.06E-02	8.97E-02	2.34E-01
	MAPE	4.6055	4.5977	5.4594	4.9171	0.0586
	r	0.9910	0.9870	0.9874	0.9983	0.9978
FT22	MAE	7.98E-02	7.75E-02	8.43E-02	9.33E-02	3.22E-01
	MAPE	4.0435	3.6742	4.0456	4.4412	0.0799
	r	0.9908	0.9868	0.9857	0.9984	0.9992

Supplementary Methods

Fast multi-locus random-SNP-effect EMMA (FASTmrEMMA)

Let $y_i (i = 1,2,\ldots,n)$ be the phenotypic value of the *i*th individual in a sample of size *n* from a natural population. The genetic model can be described as:

$$\boldsymbol{y} = \boldsymbol{W\alpha} + \boldsymbol{Z\gamma} + \boldsymbol{u} + \boldsymbol{\varepsilon} \tag{1}$$

where $\boldsymbol{y} = (y_1,\ldots,y_n)^T$; $\boldsymbol{\alpha}$ is a $c \times 1$ vector of the fixed effects, say the intercept, population structure effect and so on; $\boldsymbol{\gamma} \sim MVN_p(\boldsymbol{0}, \boldsymbol{\Sigma_\gamma})$ is QTN effects as random; $\boldsymbol{\Sigma_\gamma} = diag\{\sigma_1{}^2,\ldots,\sigma_p{}^2\}$; p is the number of putative QTNs; **W** and **Z** are the corresponding designed matrices for $\boldsymbol{\alpha}$ and $\boldsymbol{\gamma}$; polygenic effects $\boldsymbol{u} \sim MVN_n(\boldsymbol{0}, \sigma_g{}^2\boldsymbol{K})$ is a $n \times 1$ random vector; **K** is a known $n \times n$ relatedness matrix; $\boldsymbol{\varepsilon}$ is residual error with an assumed $MVN_n(\boldsymbol{0}, \sigma^2\boldsymbol{I}_n)$ distribution; σ^2 is residual error variance; $\boldsymbol{I}_n$ is a $n \times n$ identity matrix.

As $\boldsymbol{\gamma}$ is treated as being random, the variance of **y** in the model (1) is:

$$var(\boldsymbol{y}) = \boldsymbol{Z\Sigma_\gamma Z}^T + \sigma_g{}^2\boldsymbol{K} + \sigma^2\boldsymbol{I}_n = \sum_{k=1}^{p}\sigma_k{}^2\boldsymbol{Z}_k\boldsymbol{Z}_k{}^T + \sigma_g{}^2\boldsymbol{K} + \sigma^2\boldsymbol{I}_n$$

$$= \sigma^2(\sum_{k=1}^{p}\lambda_k\boldsymbol{Z}_k\boldsymbol{Z}_k{}^T + \lambda_g\boldsymbol{K} + \boldsymbol{I}_n) = \sigma^2\boldsymbol{H} \tag{2}$$

where, $\lambda_k = \sigma_k{}^2/\sigma^2 (k = 1,2,\ldots,p)$; $\lambda_g = \sigma_g{}^2/\sigma^2$; $\boldsymbol{H} = \boldsymbol{Z}diag\{\lambda_1,\ldots,\lambda_p\}\boldsymbol{Z}^T + \lambda_g\boldsymbol{K} + \boldsymbol{I}_n$.

Using Efficient Mixed Model Association (EMMA)[1], we can obtain the estimate of λ_g denoted by $\hat{\lambda}_g$. Let $\boldsymbol{B} = \hat{\lambda}_g\boldsymbol{K} + \boldsymbol{I}_n$, an eigen (or spectral) decomposition of the positive semidefinite matrix **B** is:

$$\boldsymbol{B} = \boldsymbol{Q}_B\boldsymbol{\Lambda}_B\boldsymbol{Q}_B{}^T = (\boldsymbol{Q}_1 \quad \boldsymbol{Q}_2)\begin{pmatrix}\boldsymbol{\Lambda}_r & \boldsymbol{0}\\ \boldsymbol{0} & \boldsymbol{0}\end{pmatrix}\begin{pmatrix}\boldsymbol{Q}_1{}^T\\ \boldsymbol{Q}_2{}^T\end{pmatrix}$$

$$= (\boldsymbol{Q}_1\boldsymbol{\Lambda}_r^{\frac{1}{2}}\boldsymbol{Q}_1{}^T)(\boldsymbol{Q}_1\boldsymbol{\Lambda}_r^{\frac{1}{2}}\boldsymbol{Q}_1{}^T) \quad (3)$$

Where $\boldsymbol{Q}_B$ is orthogonal, $\boldsymbol{\Lambda}_r$ is a diagonal matrix with positive eigenvalues, $r = Rank(\boldsymbol{B})$; $\mathbf{Q}_1$ and $\mathbf{Q}_2$ are the $n \times r$ and $n \times (n-r)$ block matrices of $\boldsymbol{Q}_B$; $\mathbf{0}$ is the corresponding block zero matrix[2].

Let $\boldsymbol{C} = \boldsymbol{Q}_1\boldsymbol{\Lambda}_r{}^{-\frac{1}{2}}\boldsymbol{Q}_1{}^T$, the model (1) is changed to:

$$\boldsymbol{y}_c = \boldsymbol{W}_c\boldsymbol{\alpha} + \boldsymbol{Z}_c\boldsymbol{\gamma} + \boldsymbol{\varepsilon}_c \quad (4)$$

where, $\mathbf{y}_c = \mathbf{Cy}$, $\mathbf{W}_c = \mathbf{CW}$, $\mathbf{Z}_c = \mathbf{CZ}$, $\boldsymbol{\varepsilon}_c = \mathbf{Cu} + \mathbf{C}\boldsymbol{\varepsilon} \sim MVN_n(\mathbf{0}, \sigma^2\mathbf{I}_n)$ [2].

In the model (4), let $\boldsymbol{\beta} = \binom{\boldsymbol{\alpha}}{\boldsymbol{\gamma}}$, $\boldsymbol{Y} = \boldsymbol{y}_c - \mathbf{1}_c\mu$ with a zero mean, and standardizing each column in matrix $(\boldsymbol{W}_c \quad \boldsymbol{Z}_c)$ produces a new matrix $\mathbf{X}$ with $\sum_{i=1}^{n} x_{ij} = 0$, $\sum_{i=1}^{n} x_{ij}{}^2 = 1 (j = 1,2,\ldots,p)$. Therefore, the model (4) can be rewritten as:

$$\boldsymbol{Y} = \boldsymbol{X}\boldsymbol{\beta} + \boldsymbol{\varepsilon} \quad (5)$$

Least angle regression

Assuming that $\widehat{\boldsymbol{\mu}}$ is the estimated value of the current least angle regression[3] (LARS), there are:

$$\widehat{\boldsymbol{\mu}} = \boldsymbol{X}\widehat{\boldsymbol{\beta}} = \sum_{j=1}^{k} x_j \beta_j \quad (6)$$

Let **A** be a set of indices corresponding to markers with the greatest absolute current correlations; **B** be a subset of all indices $\{1,2,\ldots,p\}$; $\boldsymbol{X_B} = \{x_1, x_2, \ldots, x_p\}$; k is the iteration steps. The specific algorithm for LARS are as follows:

Step 1: The initial value $\widehat{\boldsymbol{\mu}}(0) = \mathbf{0}$. Caculate $\boldsymbol{c} = (\boldsymbol{y} - \widehat{\boldsymbol{\mu}})^T \boldsymbol{X}$, which is the vector of current correlations. Then select the largest absolute value in **c**, $cMax = \max_j\{|c_j|\}$. Let **s** be the symbol vector of $\boldsymbol{c}$**.** Add subscript j to **A**, and remove j from **B**. Define the matrix $\boldsymbol{X_A} = (\ldots . s_j x_j \ldots .)_{j\in A}$.

Step 2: Determine the minimum angle direction $\boldsymbol{u}_A$. Let the unit column vector $1_A = (1,\ldots,1)^T$, the length of it is $|\boldsymbol{A}|$. Let $\boldsymbol{G_A} = \boldsymbol{X_A}^T \boldsymbol{X_A}, A_A = (1_A{}^T \boldsymbol{G_A}^{-1} 1_A)^{-\frac{1}{2}}$, $w_A = A_A \boldsymbol{G_A}^{-1} 1_A$, so $\boldsymbol{u}_A = \boldsymbol{X_A} w_A$ and $\boldsymbol{X_A}^T \boldsymbol{u}_A = A_A 1_A$, $||\boldsymbol{u}_A||^2 = 1$. w_A is the component of the correlation coefficient $\widehat{\boldsymbol{\beta}}$. Let $\boldsymbol{a} = \boldsymbol{X_B}^T \boldsymbol{u}_A$, which is the correlation coefficient of the minimum angle direction and gene markers in **B**.

Step 3: Calculate the step length $\hat{\gamma}$. if $|\boldsymbol{A}| = k$, $\hat{\gamma} = min^+\{\frac{cMax}{A_A}, -\frac{\widehat{\boldsymbol{\beta}}_A}{w_A \cdot \boldsymbol{s}}\}$, where min^+ means taking the smallest positive number. If $|\boldsymbol{A}| < k$, $\hat{\gamma} = min^+\{\frac{cMax - \boldsymbol{c}_B}{A_A - \boldsymbol{a}}, \frac{cMax + \boldsymbol{c}_B}{A_A + \boldsymbol{a}}, -\frac{\widehat{\boldsymbol{\beta}}_A}{w_A \cdot \boldsymbol{s}}\}$, where $\boldsymbol{c}_B$ means the correlation coefficient corresponding to $\boldsymbol{X_B}$, $\widehat{\boldsymbol{\beta}}_A$ means the regression coefficient of the gene markers corresponding to the activity set **A**.

Step 4: Update $\hat{\boldsymbol{\beta}}$ and **c**. $\hat{\boldsymbol{\beta}}$ is the regression coefficient whose each iteration is updated by weight vector w_A and step length $\hat{\gamma}$, it can be expressed as $\hat{\boldsymbol{\beta}}_A(j+1) = \hat{\boldsymbol{\beta}}_A(j) + \hat{\gamma} \cdot w_A \cdot \boldsymbol{s}$. Since the updating formula of fitting vector $\hat{\boldsymbol{\mu}}_A$ is $\hat{\boldsymbol{\mu}}(j+1) = \hat{\boldsymbol{\mu}}(j) + \hat{\gamma} \cdot \boldsymbol{u}_A$, the updating formula of **c** can be expressed as follows according to $\boldsymbol{c} = (\boldsymbol{y} - \boldsymbol{\mu})^T\boldsymbol{X}$:

$$\boldsymbol{c}(j+1) = (\boldsymbol{y} - \hat{\boldsymbol{\mu}}(j+1))^T\boldsymbol{X} = (\boldsymbol{y} - \hat{\boldsymbol{\mu}}(j) - (\hat{\boldsymbol{\mu}}(j+1) - \hat{\boldsymbol{\mu}}(j)))^T\boldsymbol{X}$$

$$= \boldsymbol{c}(j) - \hat{\gamma} \cdot \boldsymbol{u}_A{}^T\boldsymbol{X} \quad (7)$$

Step 5: When j reaches the maximum times of iterations or $|\boldsymbol{A}| = k$, stop the algorithm.

Constructing random forest models

The specific steps of the algorithm for constructing random forests[4] are as follows:

Step 1: Set the number of trees in the random forest: *ntree*. *Ntree* new self-sampling sample sets are generated from the original training dataset by using the Bagging algorithm. The samples which are not sampled at each time form *ntree* out of bag (OOB) data sets.

Step 2: One CART tree is generated by each self-sampling sample set. If there are k features, the *mtry* features are randomly extracted from the k features at each node of each tree ($mtry << k$) (generally, when dealing with regression problems, default $mtry = \frac{k}{3}$), taking the minimum node impurity, that is, the minimum Gini index, as the criterion, the feature with minimum Gini index and its corresponding segmentation point are selected from the *mtry* features, which are treated as the optimal feature and the optimal segmentation point. Then, two child nodes are generated from the current node, and the training data set is assigned to the two child nodes according to their features. Each tree grows to the maximum extent without cutting.

The Gini index of node is expressed as $Gini(t) = 1 - \sum_{j=1}^{k}[p(j|t)]^2$, k is the number of categories of test output under the current attribute, $p(j|t)$ is the probability of taking class j for sample test output in node t.

Step 3: A random forest is constructed by *ntree* CART trees. For the classification problems, random forest can classify new data with the largest number of votes as the prediction results. For the regression problems, random forests takes the average output of all CART trees as the prediction results.

Variable importance assessment of random forests

Variable importance assessment is an important feature of random forest. The importance ranking of variables are obtained by scoring the importance of variables. Random forest take the importance of variables as the variable selection standard, which increases the interpretability of the model. The variable importance measure (VIM) method for evaluating the importance of variables commonly used in random forest can be divided into two categories: one is VIM^{Gini}calculated by Gini index and the other is VIM^{OOB}calculated by OOB error rate. For the VIM^{OOB}, the classical random forest gives two importance indexes: mean decrease accuracy for classification data and *%IncMSE* for regression data. The larger the mean decrease accuracy and *%IncMSE,* the more important the variable is. For the VIM^{Gini}, the classical random forest gives two importance indexes: *mean decrease gini* for classification data and *inc node purity* for regression data. The larger the mean *decrease gini* and inc node purity, the more important the variable is. In this paper, $VIM^{OOB}$was treated as the importance scoring method and *%IncMSE* was treated as the index of importance. Assuming the sample size of the original sample set is *ntree* and the number of the variables is *k*, the generated random forest has *ntree* classification trees, that is, *ntree* OOB data sets. The specific steps of the calculation of $VIM^{OOB}$are as follows:

Step 1: For each tree classifier in a random forest, the OOB prediction error and the standard error S_E can be calculated by using the corresponding OOB data. For the classification data, the prediction error of OOB is the error rate of classification; for the regression data, the prediction error of OOB refers to MSE. The *ntree* OOB prediction errors obtained are denoted as $\%IncMSE_1, \%IncMSE_2, \ldots, \%IncMSE_k$.

Step 2: Randomly replace the value of a variable $x_i (i = 1,2,\ldots,k)$ to form new OOB test data. Take the new OOB test data to the built random forest model and calculate the prediction error under each classification tree which is denoted as $(\%IncMSE_{1i}, \%IncMSE_{2i,\ldots,}\%IncMSE_{ki})$. Thus, the matrix formed by all the OOB prediction errors is obtained as follows:

$$\begin{bmatrix} \%IncMSE_{1,1} & \%IncMSE_{1,2} & \ldots & \%IncMSE_{1,ntree} \\ \%IncMSE_{2,1} & \%IncMSE_{2,2} & \ldots & \%IncMSE_{2,ntree} \\ \ldots & \ldots & \ldots & \ldots \\ \%IncMSE_{k,1} & \%IncMSE_{k,2} & \ldots & \%IncMSE_{k,ntree} \end{bmatrix} \quad (8)$$

Step 3: For a certain prediction variable, computing its importance means the difference between the transformed prediction error and the original one. So, the importance score of x_i is expressed as:

$$score_i = \frac{VIM_i^{(OOB)}}{S_E} = \frac{\sum_{j=1}^{ntree} \frac{(\%IncMSE_j - \%IncMSE_{ij})}{ntree}}{S_E} (1 \le i \le k) \quad (9)$$

where, $S_E = \frac{\hat{\sigma}}{\sqrt{ntree}}$, $\hat{\sigma}$ is the standard error of $VIM_i^{(OOB)}$ of each tree in the random forest.

Supplementary references

1 Kang, H. M. *et al.* Efficient control of population structure in model organism association mapping. *Genetics* **178**, 1709 (2008).

2 Wen, Y.-J. *et al.* Methodological implementation of mixed linear models in multi-locus genome-wide association studies. *Briefings in Bioinformatics* **19**, 700-712, doi:10.1093/bib/bbw145 (2018).

3 Efron, B., Hastie, T., Johnstone, I. & Tibshirani, R. Least angle regression. *The Annals of Statistics* **32**, 407-451 (2004).

4 Breiman, L. Random Forests. *Machine Learning* **45**, 5-32, doi:10.1023/a:1010933404324 (2001).

Supplementary Softwares

Software S1 The program code for the two-stage algorithm based on least angle regression and random forest (TSLRF)

This file for the program code of the TSLRF includes the following files:

1) "input files”:

   Simulation analysis data:
   Supplementary Data S1.csv is genotypic values of each SNP marker for all the individuals.
   Supplementary Data S2.csv is the phenotypic values for all the individuals.

2) "program code":

The R codes for simulation analysis are as follows.

```
#### Group structure correction ####
rm(list=ls(all=T))

{
  emma.eigen.L <- function(Z,K,complete=TRUE) {
    if ( is.null(Z) ) {
      return(emma.eigen.L.wo.Z(K))
    }
    else {
      return(emma.eigen.L.w.Z(Z,K,complete))
    }
  }

  emma.eigen.L.wo.Z <- function(K) {
    eig <- eigen(K,symmetric=TRUE)
    return(list(values=eig$values,vectors=eig$vectors))
  }

  emma.eigen.L.w.Z <- function(Z,K,complete=TRUE) {
    if ( complete == FALSE ) {
      vids <- colSums(Z)>0
      Z <- Z[,vids]
      K <- K[vids,vids]
    }
    eig <- eigen(K%*%crossprod(Z,Z),symmetric=FALSE,EISPACK=TRUE)
    return(list(values=eig$values,vectors=qr.Q(qr(Z%*%eig$vectors),complete=TRUE)))
  }

  emma.eigen.R <- function(Z,K,X,complete=TRUE) {
    if ( ncol(X) == 0 ) {
      return(emma.eigen.L(Z,K))
    }
    else if ( is.null(Z) ) {
      return(emma.eigen.R.wo.Z(K,X))
    }
    else {
      return(emma.eigen.R.w.Z(Z,K,X,complete))
    }
  }
```

```
emma.eigen.R.wo.Z <- function(K, X) {
  n <- nrow(X)
  q <- ncol(X)
  S <- diag(n)-X%*%solve(crossprod(X,X))%*%t(X)
  eig <- eigen(S%*%(K+diag(1,n))%*%S,symmetric=TRUE)
  stopifnot(!is.complex(eig$values))
  return(list(values=eig$values[1:(n-q)]-1,vectors=eig$vectors[,1:(n-q)]))
}

emma.eigen.R.w.Z <- function(Z, K, X, complete = TRUE) {
  if ( complete == FALSE ) {
    vids <-  colSums(Z) > 0
    Z <- Z[,vids]
    K <- K[vids,vids]
  }
  n <- nrow(Z)
  t <- ncol(Z)
  q <- ncol(X)

  SZ <- Z - X%*%solve(crossprod(X,X))%*%crossprod(X,Z)
  eig <- eigen(K%*%crossprod(Z,SZ),symmetric=FALSE,EISPACK=TRUE)
  if ( is.complex(eig$values) ) {
    eig$values <- Re(eig$values)
    eig$vectors <- Re(eig$vectors)
  }
  qr.X <- qr.Q(qr(X))
  return(list(values=eig$values[1:(t-q)],
              vectors=qr.Q(qr(cbind(SZ%*%eig$vectors[,1:(t-q)],qr.X)),
                           complete=TRUE)[,c(1:(t-q),(t+1):n)]))
}

emma.delta.ML.LL.wo.Z <- function(logdelta, lambda, etas, xi) {
  n <- length(xi)
  delta <- exp(logdelta)
  return( 0.5*(n*(log(n/(2*pi))-1-log(sum((etas*etas)/(delta*lambda+1))))-sum(log(delta*xi+1))) )
}

emma.delta.ML.LL.w.Z <- function(logdelta, lambda, etas.1, xi.1, n, etas.2.sq ) {
  delta <- exp(logdelta)
  return( 0.5*(n*(log(n/(2*pi))-1-log(sum(etas.1*etas.1/(delta*lambda+1))+etas.2.sq))-sum(log(delta*xi.1+1)) ))

}

emma.delta.ML.dLL.wo.Z <- function(logdelta, lambda, etas, xi) {
  n <- length(xi)
  delta <- exp(logdelta)
  etasq <- etas*etas
  ldelta <- delta*lambda+1
  return( 0.5*(n*sum(etasq*lambda/(ldelta*ldelta))/sum(etasq/ldelta)-sum(xi/(delta*xi+1))) )
}

emma.delta.ML.dLL.w.Z <- function(logdelta, lambda, etas.1, xi.1, n, etas.2.sq ) {
  delta <- exp(logdelta)
  etasq <- etas.1*etas.1
  ldelta <- delta*lambda+1
  return( 0.5*(n*sum(etasq*lambda/(ldelta*ldelta))/(sum(etasq/ldelta)+etas.2.sq)-sum(xi.1/(delta*xi.1+1))) )
}

emma.delta.REML.LL.wo.Z <- function(logdelta, lambda, etas) {
  nq <- length(etas)
  delta <-  exp(logdelta)
  return( 0.5*(nq*(log(nq/(2*pi))-1-log(sum(etas*etas/(delta*lambda+1))))-sum(log(delta*lambda+1))) )
}

emma.delta.REML.LL.w.Z <- function(logdelta, lambda, etas.1, n, t, etas.2.sq ) {
  tq <- length(etas.1)
  nq <- n - t + tq
```

```
    delta <-  exp(logdelta)
    return( 0.5*(nq*(log(nq/(2*pi))-1-log(sum(etas.1*etas.1/(delta*lambda+1))+etas.2.sq))-
sum(log(delta*lambda+1))) )
  }

  emma.delta.REML.dLL.wo.Z <- function(logdelta, lambda, etas) {
    nq <- length(etas)
    delta <- exp(logdelta)
    etasq <- etas*etas
    ldelta <- delta*lambda+1
    return( 0.5*(nq*sum(etasq*lambda/(ldelta*ldelta))/sum(etasq/ldelta)-sum(lambda/ldelta)) )
  }

  emma.delta.REML.dLL.w.Z <- function(logdelta, lambda, etas.1, n, t1, etas.2.sq ) {
    t <- t1
    tq <- length(etas.1)
    nq <- n - t + tq
    delta <- exp(logdelta)
    etasq <- etas.1*etas.1
    ldelta <- delta*lambda+1
    return( 0.5*(nq*sum(etasq*lambda/(ldelta*ldelta))/(sum(etasq/ldelta)+etas.2.sq)-sum(lambda/ldelta) ))
  }

  emma.MLE <- function(y, X, K, Z=NULL, ngrids=100, llim=-10, ulim=10,
                     esp=1e-10, eig.L = NULL, eig.R = NULL)
  {
    n <- length(y)
    t <- nrow(K)
    q <- ncol(X)

    stopifnot(ncol(K) == t)
    stopifnot(nrow(X) == n)

    if ( det(crossprod(X,X)) == 0 ) {
      warning("X is singular")
      return (list(ML=0,delta=0,ve=0,vg=0))
    }

    if ( is.null(Z) ) {
      if ( is.null(eig.L) ) {
        eig.L <- emma.eigen.L.wo.Z(K)
      }
      if ( is.null(eig.R) ) {
        eig.R <- emma.eigen.R.wo.Z(K,X)
      }
      etas <- crossprod(eig.R$vectors,y)


      logdelta <- (0:ngrids)/ngrids*(ulim-llim)+llim
      m <- length(logdelta)
      delta <- exp(logdelta)

      Lambdas.1<-matrix(eig.R$values,n-q,m)
      Lambdas <- Lambdas.1 * matrix(delta,n-q,m,byrow=TRUE)+1
      Xis.1<-matrix(eig.L$values,n,m)
      Xis <- Xis.1* matrix(delta,n,m,byrow=TRUE)+1
      Etasq <- matrix(etas*etas,n-q,m)
      dLL <- 0.5*delta*(n*colSums(Etasq*Lambdas.1/(Lambdas*Lambdas))/colSums(Etasq/Lambdas)-
colSums(Xis.1/Xis))
      optlogdelta <- vector(length=0)
      optLL <- vector(length=0)
      if ( dLL[1] < esp ) {
        optlogdelta <- append(optlogdelta, llim)
        optLL <- append(optLL, emma.delta.ML.LL.wo.Z(llim,eig.R$values,etas,eig.L$values))
      }
      if ( dLL[m-1] > 0-esp ) {
        optlogdelta <- append(optlogdelta, ulim)
```

```
      optLL <- append(optLL, emma.delta.ML.LL.wo.Z(ulim,eig.R$values,etas,eig.L$values))
    }

    for( i in 1:(m-1) )
    {
      if ( ( dLL[i]*dLL[i+1] < 0-esp*esp ) && ( dLL[i] > 0 ) && ( dLL[i+1] < 0 ) )
      {
        r <- uniroot(emma.delta.ML.dLL.wo.Z, lower=logdelta[i], upper=logdelta[i+1], lambda=eig.R$values,
etas=etas, xi=eig.L$values)
        optlogdelta <- append(optlogdelta, r$root)
        optLL <- append(optLL, emma.delta.ML.LL.wo.Z(r$root,eig.R$values, etas, eig.L$values))
      }
    }
  }
  else {
    if ( is.null(eig.L) ) {
      eig.L <- emma.eigen.L.w.Z(Z,K)
    }
    if ( is.null(eig.R) ) {
      eig.R <- emma.eigen.R.w.Z(Z,K,X)
    }
    etas <- crossprod(eig.R$vectors,y)
    etas.1 <- etas[1:(t-q)]
    etas.2 <- etas[(t-q+1):(n-q)]
    etas.2.sq <- sum(etas.2*etas.2)

    logdelta <- (0:ngrids)/ngrids*(ulim-llim)+llim

    m <- length(logdelta)
    delta <- exp(logdelta)

    Lambdas.1<-matrix(eig.R$values,t-q,m)
    Lambdas <- Lambdas.1 * matrix(delta,t-q,m,byrow=TRUE) + 1

    Xis.1<-matrix(eig.L$values,t,m)
    Xis <- Xis.1 * matrix(delta,t,m,byrow=TRUE) + 1
    Etasq <- matrix(etas.1*etas.1,t-q,m)

    dLL <- 0.5*delta*(n*colSums(Etasq*Lambdas.1/(Lambdas*Lambdas))/(colSums(Etasq/Lambdas)+etas.2.sq)-
colSums(Xis.1/Xis))
    optlogdelta <- vector(length=0)
    optLL <- vector(length=0)
    if ( dLL[1] < esp ) {
      optlogdelta <- append(optlogdelta, llim)
      optLL <- append(optLL, emma.delta.ML.LL.w.Z(llim,eig.R$values,etas.1,eig.L$values,n,etas.2.sq))
    }
    if ( dLL[m-1] > 0-esp ) {
      optlogdelta <- append(optlogdelta, ulim)
      optLL <- append(optLL, emma.delta.ML.LL.w.Z(ulim,eig.R$values,etas.1,eig.L$values,n,etas.2.sq))
    }

    for( i in 1:(m-1) )
    {
      if ( ( dLL[i]*dLL[i+1] < 0-esp*esp ) && ( dLL[i] > 0 ) && ( dLL[i+1] < 0 ) )
      {
        r <- uniroot(emma.delta.ML.dLL.w.Z, lower=logdelta[i], upper=logdelta[i+1], lambda=eig.R$values,
etas.1=etas.1, xi.1=eig.L$values, n=n, etas.2.sq = etas.2.sq )
        optlogdelta <- append(optlogdelta, r$root)
        optLL <- append(optLL, emma.delta.ML.LL.w.Z(r$root,eig.R$values, etas.1, eig.L$values, n, etas.2.sq ))
      }
    }
  }

  maxdelta <- exp(optlogdelta[which.max(optLL)])
  maxLL <- max(optLL)
  if ( is.null(Z) ) {
    maxve <- sum(etas*etas/(maxdelta*eig.R$values+1))/n
```

```
    }
    else {
      maxve <- (sum(etas.1*etas.1/(maxdelta*eig.R$values+1))+etas.2.sq)/n
    }
    maxvg <- maxve*maxdelta

    return (list(ML=maxLL,delta=maxdelta,ve=maxve,vg=maxvg))
  }

  emma.REMLE <- function(y, X, K, Z=NULL, ngrids=100, llim=-10, ulim=10,
                         esp=1e-10, eig.L = NULL, eig.R = NULL) {
    n <- length(y)
    t <- nrow(K)
    q <- ncol(X)

    stopifnot(ncol(K) == t)
    stopifnot(nrow(X) == n)

    if ( det(crossprod(X,X)) == 0 ) {
      warning("X is singular")
      return (list(REML=0,delta=0,ve=0,vg=0))
    }

    if ( is.null(Z) ) {
      if ( is.null(eig.R) ) {
        eig.R <- emma.eigen.R.wo.Z(K,X)
      }
      etas <- crossprod(eig.R$vectors,y)

      logdelta <- (0:ngrids)/ngrids*(ulim-llim)+llim
      m <- length(logdelta)
      delta <- exp(logdelta)

      Lambdas.1<-matrix(eig.R$values,n-q,m)
      Lambdas <- Lambdas.1 * matrix(delta,n-q,m,byrow=TRUE) + 1
      Etasq <- matrix(etas*etas,n-q,m)

      dLL <- 0.5*delta*((n-q)*colSums(Etasq*Lambdas.1/(Lambdas*Lambdas))/colSums(Etasq/Lambdas)-
colSums(Lambdas.1/Lambdas))

      optlogdelta <- vector(length=0)
      optLL <- vector(length=0)
      if ( dLL[1] < esp ) {
        optlogdelta <- append(optlogdelta, llim)
        optLL <- append(optLL, emma.delta.REML.LL.wo.Z(llim,eig.R$values,etas))
      }
      if ( dLL[m-1] > 0-esp ) {
        optlogdelta <- append(optlogdelta, ulim)
        optLL <- append(optLL, emma.delta.REML.LL.wo.Z(ulim,eig.R$values,etas))
      }

      for( i in 1:(m-1) )
      {
        if ( ( dLL[i]*dLL[i+1] < 0-esp*esp ) && ( dLL[i] > 0 ) && ( dLL[i+1] < 0 ) )
        {
          r <- uniroot(emma.delta.REML.dLL.wo.Z, lower=logdelta[i], upper=logdelta[i+1], lambda=eig.R$values,
etas=etas)
          optlogdelta <- append(optlogdelta, r$root)
          optLL <- append(optLL, emma.delta.REML.LL.wo.Z(r$root,eig.R$values, etas))
        }
      }
    }
    else {
      if ( is.null(eig.R) ) {
        eig.R <- emma.eigen.R.w.Z(Z,K,X)
      }
      etas <- crossprod(eig.R$vectors,y)
```

```
      etas.1 <- etas[1:(t-q)]
      etas.2 <- etas[(t-q+1):(n-q)]
      etas.2.sq <- sum(etas.2*etas.2)

      logdelta <- (0:ngrids)/ngrids*(ulim-llim)+llim
      m <- length(logdelta)
      delta <- exp(logdelta)

      Lambdas.1 <- matrix(eig.R$values,t-q,m)
      Lambdas <- Lambdas.1 * matrix(delta,t-q,m,byrow=TRUE) + 1
      Etasq <- matrix(etas.1*etas.1,t-q,m)

      dLL <- 0.5*delta*((n-q)*colSums(Etasq*Lambdas.1/(Lambdas*Lambdas))/(colSums(Etasq/Lambdas)+etas.2.sq)-
colSums(Lambdas.1/Lambdas))

      optlogdelta <- vector(length=0)
      optLL <- vector(length=0)
      if ( dLL[1] < esp ) {
        optlogdelta <- append(optlogdelta, llim)
        optLL <- append(optLL, emma.delta.REML.LL.w.Z(llim,eig.R$values,etas.1,n,t,etas.2.sq))
      }
      if ( dLL[m-1] > 0-esp ) {
        optlogdelta <- append(optlogdelta, ulim)
        optLL <- append(optLL, emma.delta.REML.LL.w.Z(ulim,eig.R$values,etas.1,n,t,etas.2.sq))
      }

      for( i in 1:(m-1) )
      {
        if ( ( dLL[i]*dLL[i+1] < 0-esp*esp ) && ( dLL[i] > 0 ) && ( dLL[i+1] < 0 ) )
        {
          r <- uniroot(emma.delta.REML.dLL.w.Z, lower=logdelta[i], upper=logdelta[i+1], lambda=eig.R$values,
etas.1=etas.1, n=n, t1=t, etas.2.sq = etas.2.sq )
          optlogdelta <- append(optlogdelta, r$root)
          optLL <- append(optLL, emma.delta.REML.LL.w.Z(r$root,eig.R$values, etas.1, n, t, etas.2.sq ))
        }
      }
    }

    maxdelta <- exp(optlogdelta[which.max(optLL)])
    maxLL <- max(optLL)

    if ( is.null(Z) ) {
      maxve <- sum(etas*etas/(maxdelta*eig.R$values+1))/(n-q)
    }
    else {
      maxve <- (sum(etas.1*etas.1/(maxdelta*eig.R$values+1))+etas.2.sq)/(n-q)
    }
    maxvg <- maxve*maxdelta
    return (list(REML=maxLL,delta=maxdelta,ve=maxve,vg=maxvg))
  }

  emma.maineffects.B<-function(Z=NULL,K,deltahat.g,complete=TRUE){
    if( is.null(Z) ){
      return(emma.maineffects.B.Zo(K,deltahat.g))
    }
    else{
      return(emma.maineffects.B.Z(Z,K,deltahat.g,complete))
    }
  }

  emma.maineffects.B.Zo <-function(K,deltahat.g){
    t <- nrow(K)
    stopifnot(ncol(K) == t)

    B<-deltahat.g*K+diag(1,t)
    eig<-eigen(B,symmetric=TRUE)
    qr.B<-qr(B)
```

```
    q<-qr.B$rank

    stopifnot(!is.complex(eig$values))

    A<-diag(1/sqrt(eig$values[1:q]))
    Q<-eig$vectors[,1:q]
    C<-Q%*%A%*%t(Q)
    return(list(mC=C,Q=Q,A=A))
  }

  emma.maineffects.B.Z <- function(Z,K,deltahat.g,complete=TRUE){
    if ( complete == FALSE ) {
      vids <- colSums(Z)>0
      Z <- Z[,vids]
      K <- K[vids,vids]
    }

    n <- nrow(Z)
    B <- deltahat.g*Z%*%K%*%t(Z)+diag(1,n)
    eig <- eigen(B,symmetric=TRUE,EISPACK=TRUE)
    qr.B<-qr(B)
    q<-qr.B$rank

    stopifnot(!is.complex(eig$values))

    A<-diag(1/sqrt(eig$values[1:q]))
    Q<-eig$vectors[,1:q]
    C<-Q%*%A%*%t(Q)
    return(list(mC=C,Q=Q,A=A,complete=TRUE))
  }

  emma.MLE0.c <- function(Y_c,W_c){

    n <- length(Y_c)

    stopifnot(nrow(W_c)==n)

    M_c<-diag(1,n)-W_c%*%solve(crossprod(W_c,W_c))%*%t(W_c)
    etas<-crossprod(M_c,Y_c)

    LL <- 0.5*n*(log(n/(2*pi))-1-log(sum(etas*etas)))
    return(list(ML=LL))

  }

  emma.REMLE0.c <- function(Y_c,W_c){

    n <- length(Y_c)

    stopifnot(nrow(W_c)==n)

    M_c <-diag(1,n)-W_c%*%solve(crossprod(W_c,W_c))%*%t(W_c)
    eig <-eigen(M_c)
    t <-qr(W_c)$rank
    v <-n-t
    U_R <-eig$vector[,1:v]
    etas<-crossprod(U_R,Y_c)

    LL <- 0.5*v*(log(v/(2*pi))-1-log(sum(etas*etas)))
    return(list(REML=LL))
  }
}


####initialization####
dir <-"D:/"
dirout <- "D:/"
```

```
setwd(dir)
time <- numeric()
x.data <- as.matrix(read.csv("xx_all.csv",header=F))
y.data <- as.matrix(read.csv("yy_or1.csv",header=F))
nsam <-ncol(x.data)-2
nmak <- nrow(x.data)
nrep <- 1000
nrepl <- 1000
i <- 1
ntree_set <- 500
mtry_set <- 11
chrnum<-length(unique(x.data[,1]))
tt0 <- proc.time()
larsall <- numeric()
imp <- matrix(0,nsam,3*nrep)
cor_matrix <- matrix(0,1,nrep)
library(lars)
library(randomForest)
####calculate importance scores of simulation datasets of 1000 replications using TSLRF####
  for(repl in 1:nrepl){
    tt1 <- proc.time()
    res0 <- numeric()
    larsres <- numeric()
    print(repl)

    ####calculate K from other Chromosomes####
    xxot <- as.matrix(x.data[,3:ncol(x.data)])
    xot <-t(xxot)
    nmarkot <-ncol(xot)
    kk<-matrix(0,nsam,nsam)
    for(k in 1:nmarkot){
      z<-as.matrix(xot[,k])
      kk<-kk+z%*%t(z)}
    cc<-mean(diag(kk))
    K <- numeric()
    K <- kk/cc
    ####finish####

    xx <- as.matrix(x.data[,3:ncol(x.data)])
    YY <- matrix(y.data,,nrep)
    W.orig<-matrix(1,nsam,1)
    W <- W.orig

    ####the population structure and polygenic background controls####
    remle2<-emma.REMLE(YY[,repl], W, K, Z=NULL, ngrids=100, llim=-10, ulim=10,esp=1e-10, eig.L = NULL,
eig.R = NULL)
    remle1.B1<-emma.maineffects.B(Z=NULL,K,remle2$delta)
    C2<-remle1.B1$mC
    ####finish####

    y <- as.matrix(C2%*%YY[,repl])
    x <- cbind(C2%*%W,C2%*%t(xx))
 ####parameter estimation by LARS####
  LAR <- lars(x,y,type="lar",trace = FALSE, normalize = TRUE, intercept = TRUE,use.Gram=F,max.steps=198)
  res_order <- which((LAR$beta[nrow(LAR$beta),])!=0)
  chr <- x.data[res_order,1:2]
  a <- seq(1,10000,length=10000)
  b <- a[res_order]
 ####finish####

 ####calculate importance scores of the parameters selected by LARS using RF####
  geno.rf <- randomForest(x[,res_order],as.vector(y),ntree=ntree_set,mtry=mtry_set,importance=T)
  pred <- predict(geno.rf,x[,res_order],type="response")
  cor_matrix[,repl] <- cor(pred,y,method = "pearson")
  imn <- order(importance(geno.rf)[,1],decreasing=T)
  ims <- sort(importance(geno.rf)[,1],decreasing=T)
  imp[1:length(res_order),i:(i+2)] <- c(chr[imn,],ims)
```

```
  i <- i+3
}
tt1 <- proc.time()-as.matrix(tt0)

####Export results####
write.table(tt1,"D:/TSLRF-time-1000.csv",sep=",")
write.table(imp,"D:/TSLRF-importance score-1000.csv",sep=",",row.names = FALSE)
write.table(cor_matrix,"D://TSLRF-pearson-1000.csv",sep=",",row.names = FALSE)
```
